# Mining thermophiles for biotechnologically relevant enzymes: evaluating the potential of European and Caucasian hot springs

**DOI:** 10.1007/s00792-023-01321-3

**Published:** 2023-11-22

**Authors:** Christin Burkhardt, Leon Baruth, Neele Meyer-Heydecke, Barbara Klippel, Armine Margaryan, Ani Paloyan, Hovik H. Panosyan, Garabed Antranikian

**Affiliations:** 1 Institute of Technical Biocatalysis, Center for Biobased Solutions, Hamburg University of Technology, Am Schwarzenberg-Campus 4, 21073 Hamburg, Germany; 2 Department of Biochemistry, Microbiology and Biotechnology, Yerevan State University, Alex Manoogian 1, 0025 Yerevan, Armenia; 3 Research Institute of Biology, Yerevan State University, Alex Manoogian 1, 0025 Yerevan, Armenia; 4 Scientific and Production Center, “Armbiotechnology” NAS RA, 14 Gyurjyan Str. 0056, Yerevan, Armenia

**Keywords:** Extremophiles, Hot spring, Europe, Caucasus, Enzymes, Bidiversity

## Abstract

The development of sustainable and environmentally friendly industrial processes is becoming very crucial and demanding for the rapid implementation of innovative bio-based technologies. Natural extreme environments harbor the potential for discovering and utilizing highly specific and efficient biocatalysts that are adapted to harsh conditions. This review focuses on extremophilic microorganisms and their enzymes (extremozymes) from various hot springs, shallow marine vents, and other geothermal habitats in Europe and the Caucasus region. These hot environments have been partially investigated and analyzed for microbial diversity and enzymology. Hotspots like Iceland, Italy, and the Azores harbor unique microorganisms, including bacteria and archaea. The latest results demonstrate a great potential for the discovery of new microbial species and unique enzymes that can be explored for the development of Circular Bioeconomy.

Different screening approaches have been used to discover enzymes that are active at extremes of temperature (up 120 °C), pH (0.1 to 11), high salt concentration (up to 30%) as well as activity in the presence of solvents (up to 99%). The majority of published enzymes were revealed from bacterial or archaeal isolates by traditional activity-based screening techniques. However, the latest developments in molecular biology, bioinformatics, and genomics have revolutionized life science technologies. Post-genomic era has contributed to the discovery of millions of sequences coding for a huge number of biocatalysts. Both strategies, activity- and sequence-based screening approaches, are complementary and contribute to the discovery of unique enzymes that have not been extensively utilized so far.

## Introduction

Due to many advantages, biocatalysts are becoming more and more attractive for the development of sustainable and innovative biotechnological processes. Being highly specific and efficient, this transformation will contribute to the implementation of “greener” and sustainable industrial processes. This environmentally friendly approach is crucial for the development of Circular Bioeconomy which will find application in many fields including food, feed, textile, pharmaceutical, health, and renewable energy (Antranikian and Streit 2022). The enzymes of interest include all seven enzyme classes: oxidoreductases (EC 1) transferases (EC 2), hydrolases (EC 3), lyases (EC 4), isomerases (EC 5), ligases (EC 6), and translocases (EC 7). A number of representatives of these classes have been shown to be able to increase the turnover rate in second- and third-generation biorefineries (Zuliani et al. 2021), underlining the need for the discovery of novel enzymes. Future demand for enzymes for example comprises in fields of sustainable material recycling (e.g., PET degradation, Wei et al. 2019), medical science (e.g., biofilm deconstruction, Costa et al. 2020) or de novo synthesis of biomolecules with complex enzyme cascades (Yi et al. 2021).

Especially enzymes derived from extremophilic microorganisms (extremozymes) are attractive candidates for industrial processes that run under extreme conditions such as high temperatures, high pressure, high solvent concentration, high salinity, and at extremes of pH (Elleuche et al. 2014; Krüger et al. 2018; Singh and Ray 2021). Beside higher activity and stability under harsh conditions, extremozymes offer further attractive properties like simplified purification and better productivity (Siddiqui 2015). In the last five decades, a number of unique enzymes from extremophiles has been studied in detail and showed activities between − 10 and 120 °C and pH 0.2 to 12. One of the most fascinating group of microorganisms are thermophiles, including bacteria and archaea, that can survive at elevated temperatures from 50 up to 120 °C. They are present worldwide in high-temperature environments, which are caused by natural geothermal processes or man-made conditions (e.g., hot water tubes, compost). Many of these habitats are geothermal springs, which are quite diverse and appear as water springs, geysers or fumaroles in terrestrial, shallow marine or deep sea habitats.

In Europe and Caucasus region, such environments are spread over the whole continent. There are hotspots in separated insularity on the mid-Atlantic ridge (Iceland, Azores) as well as geothermal sites on the coastline of the Mediterranean Sea (Italy, Greece) and central locations (Portugal, Spain, Caucasus mountains). In the last 50 years, archaea as well as bacteria have been isolated by classical enrichment methods. Bacteria being the majority probably due to the challenging cultivation of archaea (Suleiman et al. 2020). The latest development in molecular biology and bioinformatics including metagenomics, metatranscriptomics, and metabolomics contributed significantly to the exploration of such habitats in detail shedding light on biodiversity, metabolic pathways, and enzymology.

Although many microbial species of hot springs seem to be globally distributed, isolated terrestrial habitats can lead to diversification of the genomes in correlation to the geographic distance (Erikstad et al. 2019). Therefore, investigation of different hot spring habitats can reveal multiple enzyme variants which were selected and improved by nature during evolution. The obtained sequence and structure variations are useful to understand their effects on the enzyme properties. This knowledge advances rational design in enzyme engineering and will contribute to design enzymes with desired properties for various applications (Watanabe et al. 2018; Ali et al. 2020).

In this review, we will give an overview on the distribution of hot springs (above 50 °C) in Europe and Caucasus region and present information regarding their biodiversity and enzymology, which is of relevance to biotechnological processes. We will present the variety of methods applied in screening for novel thermostable enzymes and highlight the current trends and future perspectives.

## Hot springs

Life is able to thrive under a variety of extreme conditions. Hot springs can be found all over the world and are the breeding pool for life itself (Des Marais and Walter 2019)**.** They form when groundwater is geothermally heated by the Earth's crust and rises to the surface. This process occurs in volcanic areas and also non-volcanic areas, by contact with magma or geothermal heat from the Earth’s interior, respectively (Mehta and Satyanarayana 2013). Extensive mineralization occurs while the heated water is ascending through the Earth and dissolving minerals on its way, leading to a saturation of different minerals, including H_2_S, CO_2_, CH_4_, H_2_, NH_3_, low-molecular-weight compounds, and trace elements (Brock 1978). In addition, reduced chemical compounds and other solutes are delivered to more oxidized surface environments, providing redox energy and nutrients (Des Marais and Walter 2019). Thus, diverse chemical compositions and thermal gradients are created and are able to harbor a great diversity and abundance of microbial communities. Depending on the dissolved minerals and other solutes, the pH of a hot spring can vary from pH 0.1 to pH 11, each with their own dominating organisms (Dodds and Whiles 2010).

Besides hot water springs, other types of springs can be found. A geyser is a special kind of terrestrial hot spring which can generally be found near active volcanic areas (Mehta and Satyanarayana 2013). Surface water moves down and comes in contact with hot rocks at a depth of around 2000 m, leading to boiling of the pressurized water. Water and steam are then ejected through the geyser´s surface vent and can exceed heights of 115 m (Dodds and Whiles 2010; Reed et al. 2021).

Another kind of terrestrial hot spring is the fumarole. A fumarole is a steam vent that emerges through an opening in the Earth’s crust like cracks or long fissures, emitting steam and gasses (i.e., CO_2_, SO_2_, HCl, H_2_S), as well as on the surface of lava flows (Satyanarayana et al. 2013). If a fumarole emits sulfurous gasses, it is called a solfatara and can be found within so-called solfataric fields. Magma chambers located beneath these soils, mud pots, and sulfur-rich springs heat up these solfatara to temperatures of up to 100 °C (Satyanarayana et al. 2013). Black smokers are marine hydrothermal fumaroles able to heat the surrounding water above 400 °C (Stetter; Colín-García, 2016). These hydrothermal vents are a part of a subaqueous hydrothermal system which, much like terrestrial hot springs, are rich in nutrients and are also considered to be a potential cradle for the origin of life and can be found in the deep sea along mid ocean ridges (Mehta and Satyanarayana 2013; Colín-García, 2016; Des Marais and Walter 2019). Marine vents can also occur in shallow waters close to the shore, like at Vulcano Island (Italy), where they exhibit temperatures of 80–105 °C at depths of 1–10 m (Stetter 2001). Depending on the tide, some of these shallow marine vents can lie dry or below the water surface, showing marine and terrestrial properties alike.

Terrestrial hot springs can be found all over the world, with the most prominent sites being Iceland, New Zealand, Chile, Japan and specifically Kamchatka (Russia) and the Yellowstone National Park (Wyoming, USA) (Satyanarayana et al. 2013). In 1969, Brock and Freeze isolated the thermophile *Thermus aquaticus* from a hot spring in Yellowstone National Park, setting the starting point for research on thermophilic extremophiles (Brock and Freeze 1969). Following this pioneering research, the understanding of the upper temperature limit for life grew, as Fiala and Stetter isolated *Pyrococcus furiosus* from geothermally heated marine sediment, an archaeon with an optimal temperature of growth at 100 °C (Fiala and Stetter 1986). *Pyrolobus fumarii*, isolated from a black smoker wall, exhibited an even higher temperature of growth with 113 °C (Blöchl et al. 1997). However, the archaeon “strain 121”, also isolated from a black smoker, is able to grow at a temperature of 121 °C, defining the upper limit for life to date (Kashefi and Lovley 2003).

## Strategies for enzyme screening

**Fig. 1:**
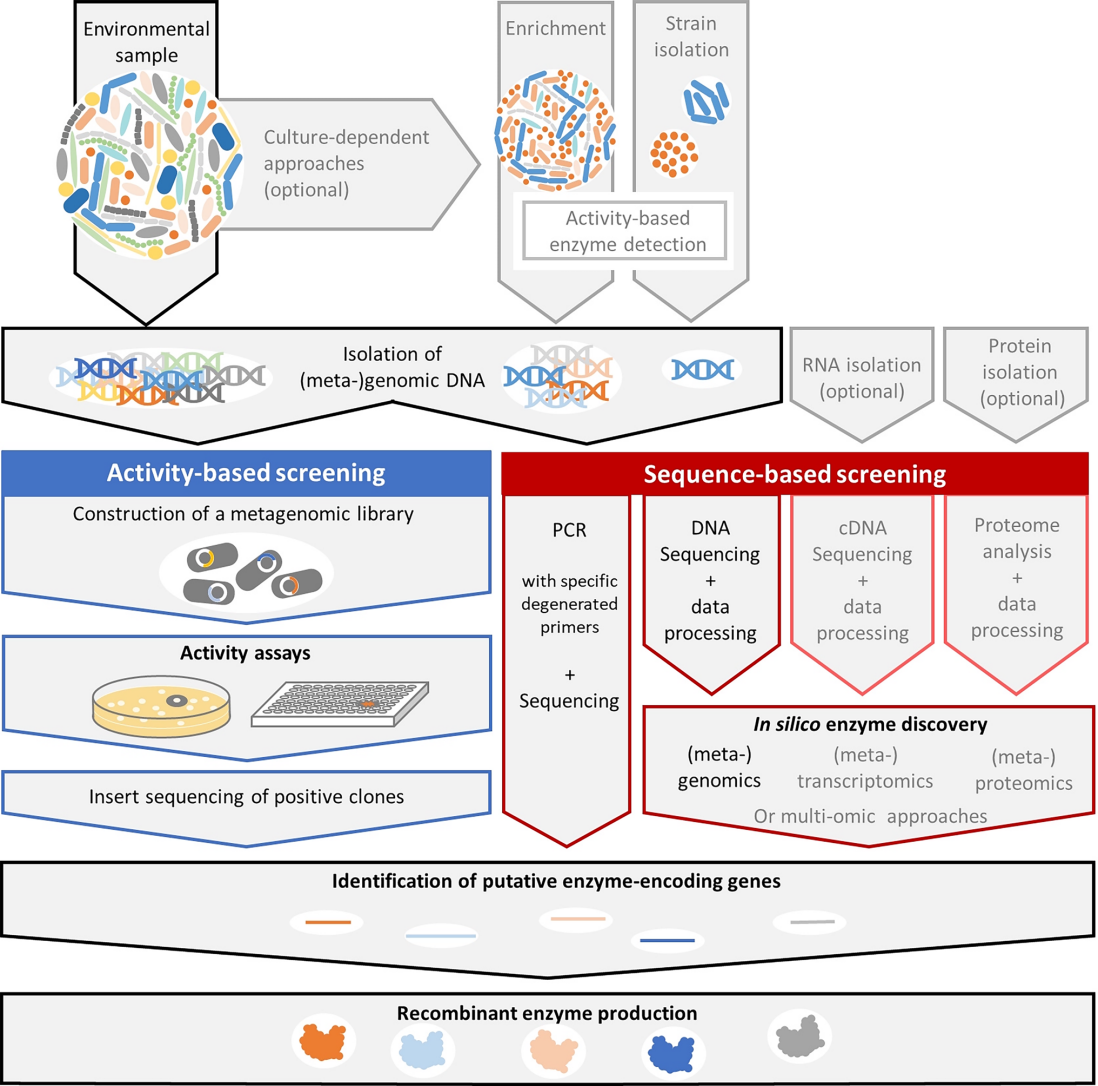
Overview of different strategies for activity-based and sequence-based screening of enzymes starting from environmental samples

Thermophilic microorganisms from hot springs are a valuable source of stable enzymes for biotechnological applications. For the identification of these enzymes, versatile approaches have been developed in the last decades (Fig. 1). Enzymes were discovered directly from the isolated microorganisms itself or by screening for enzyme-encoding genes. The latter technique was a milestone for recombinant enzyme production in optimized expression strains like *E. coli* as well as for understanding of their sequence and function relationship. Screening for enzyme-encoding genes can be achieved by different approaches, optionally including enrichment strategies followed by either activity-based or sequence-based screening methods. According to different publication analyses, the vast majority of enzymes in the past was screened by function-based screening approaches (Ferrer et al. 2016; Berini et al. 2017). Nevertheless, in the last few years, function-based screening is increasingly replaced by the cheaper and even faster sequence-based approaches (Kelly et al. 2020). The starting point for all these strategies is isolated DNA either from a single species (genomic DNA) or from a microbial community of an environmental sample or enrichment cultures (metagenomic DNA). In addition, RNA for (meta-)transcriptome analysis or proteins for (meta-)proteome analysis can be used as well.

## Enrichment and strain isolation

This classical approach has been implemented for more than a half-century and led to the discovery of unique microorganisms, metabolic pathways, and enzymes. An enrichment culture of an environmental sample can support the growth of suitable microorganisms found in a consortium. Samples are taken from an environment and incubated in a medium containing growth requiring nutrients. Moreover, the cultivation of aerobic and anaerobic microorganism requires special methods and media. By adding a selected substrate, specialized microorganisms are enriched. Beside the classical cultivation of microorganisms in laboratory, in situ enrichments are an attractive strategy. Hereby, the desired substrates and an inoculum are within a membrane locked compartment, which is directly added to the hot spring sampling sites to enable exchange of minerals and conditions. Such in situ enrichments were performed, for example, in two hot springs in Iceland with starch prior to screening of starch- and pullulan-modifying enzymes (Labes et al. 2008) as well as with diverse biopolymers in Russia and Iceland for screening of thermostable hydrolases (Wohlgemuth et al. 2018).

As shown in Fig. 1, an enrichment culture can be further treated and isolation of pure cultures can be achieved by classical microbiology methods. In the beginning of the era of enzyme discovery, classical enrichment and isolation followed by activity assays were the key strategies for identifying enzyme-producing microorganisms. This approach has been successful in the discovery of various classes of heat stable enzymes including carbohydrases, proteases, and lipases in the last decades. With plant polymers as substrates, microbial species producing thermostable enzymes like cellulases, xylanases, and oxidoreductases were identified.

However, recombinant production or enzyme improvement require the corresponding gene sequences. In this regard, additional activity-based screening of (meta-)genomic libraries or sequence-based screening techniques are necessary. Therefore, enrichment strategies are often applied prior to activity- or sequence-based screening of enzyme-encoding genes. By this, they can increase screening success and reduce the screening costs and effort. Moreover, they can be useful when target substrates occur in low concentrations in nature or for hardly degradable substrates. Recently, this approach was also successful for the enrichment of microorganisms capable of metabolizing the hardly biodegradable PET which was the origin for the identification of PET-degrading enzyme classes (Yoshida et al. 2016).

## Activity-based screening

Activity-based screening of thermophilic isolates marked the beginning of the discovery and characterization of thermostable enzymes. For decades, this strategy was state of the art and during this time, many of the fundamental concepts of plate-based activity assays were developed. Well-established and common assays utilize dye-coupled polymers (e.g., polysaccharides and polypeptides) or pH shifts. These techniques provided a basis for the later emerging screening of (meta-)genomic libraries as well. (Meta)-genomic libraries are necessary for identifying the enzyme-encoding genes. The encoding genes are required for the heterologous overexpression in production hosts (e.g., *E. coli*, *Bacillus* sp.) as well as to determine sequence motifs, domain structures, and an enzyme classification system.

For constructing (meta)-genomic libraries, genomic or metagenomic DNA has to be isolated, fragmented and subcloned into appropriate vectors. Plasmids, fosmids, phagemid, cosmid, or BAC-based systems are available whereas fosmid and plasmid libraries are often preferred (Berini et al. 2017). Vectors have then to be transferred to a screening host to induce gene expression whereby almost exclusively *E. coli* is used. Typical bottlenecks like promoter recognition, codon usage, and proper folding for enzyme activation have led to alternative expression systems like *Thermus thermophilus* (Angelov et al. 2009; Leis et al. 2015).

The main advantage of activity-based screening is the identification of totally new enzyme classes or enzymes with unknown domain structures. Knowledge of sequences or protein structures is not required, though sensitive and selective intelligent screening assays have to be available. Beside plate-based activity screening of (meta-)genomic libraries, microtiter plate-based liquid assays, which are photometrically accessible, have been successfully applied. Multiple assays in parallel, robotics, and/or cell-free expression can increase hit rates and minimize material input and time (Maruthamuthu et al. 2016; Dixit et al. 2021; Rolf et al. 2019; Markel et al. 2020). Challenges with novel target substrates like, for example, artificial plastic polymers are mastered with model substrates like oligomers or functionalized esters for PET degradation (Charnock 2021; Pirillo et al. 2021).

## Sequence-based screening approaches

This approach focuses on DNA sequences that are generated by sequencing of pure microbial strains, mixed cultures or environmental samples. DNA and RNA can be isolated from these samples without an active microbial population. From this data, information regarding the biodiversity in different habitats and enzymology can be analyzed in detail.

With an increasing number of enzyme sequences in databases and improved prediction tools, sequence-based screening approaches are advancing dramatically. The first approaches started with degenerated primers based on sequence alignments with subsequent PCR and genome walking (Kotik 2009). This was an expedient but laborious strategy for creating a collection of similar genes which is nowadays almost completely replaced by next generation sequencing methods. For this, a tool box has been developed which could be adapted to the particular environment and screening approach. A comprehensive overview of the principles of library preparation (DNA extraction, fragmentation, adapter insertion), sequencing platforms (Roche 454, Illumina, AB SOLiD, Helicos Biosciences, Nanopore MinION, PacBio), and tools for quality control, genome assembly as well as taxonomic and functional annotation is given by Almeida and Martinis (2019). A detailed comparison of tools and strategies for sequence data analysis is reported by Pal et al. (2018) and Robinson et al. (2021).

Compared to the activity-based screening approaches mentioned above, sequence-based screening can reach a much higher number of putative enzyme candidates in shorter times (Ferrer et al. 2016). Preselection processes need to be applied like in silico function and structure prediction or even a combination with activity-based approaches to identify the most promising enzyme candidates. Multi- “omic” approaches can overcome limitations in activity prediction and the enormous number of putative enzyme candidates (Krüger et al. 2020). By combining data from metagenomics, metatranscriptomics, or metaproteomics, more detailed information regarding biodiversity and enzymology of microbial communities can be presented. Such a strategy has been already applied for biogas plant analysis (Bremges et al. 2015) and analysis of hot spring communities (Busch et al. 2021).

The bottleneck is the successful production of recombinant enzymes in expression strains such as *E. coli*, *Bacillus sp.* or yeasts. Gene synthesis can overcome codon usage limitations whereas proper folding of proteins from thermophiles can be improved by thermophilic expression strains like *Thermus thermophilus* (Moreno et al. 2003; Fujino et al. 2020), *Saccharolobus solfataricus* (Albers et al. 2006), *Sulfolobus acidocaldarius* (Suzuki and Kurosawa 2016) or *Geobacillus thermodenitrificans* (Daas et al. 2018).

## Screening of hot springs in Europe and Caucasus

Hot springs caused by geothermal activities are spread worldwide. Like other continents, Europe and Caucasus regions harbor a vast abundance of terrestrial hot springs that have been analyzed to gain fundamental knowledge in microbiology by isolation of strains or metagenomic analysis. Accompanied with that, the screening of thermoactive enzymes was performed in parallel or based on the microbial isolates or metagenomic datasets.

**Fig. 2:**
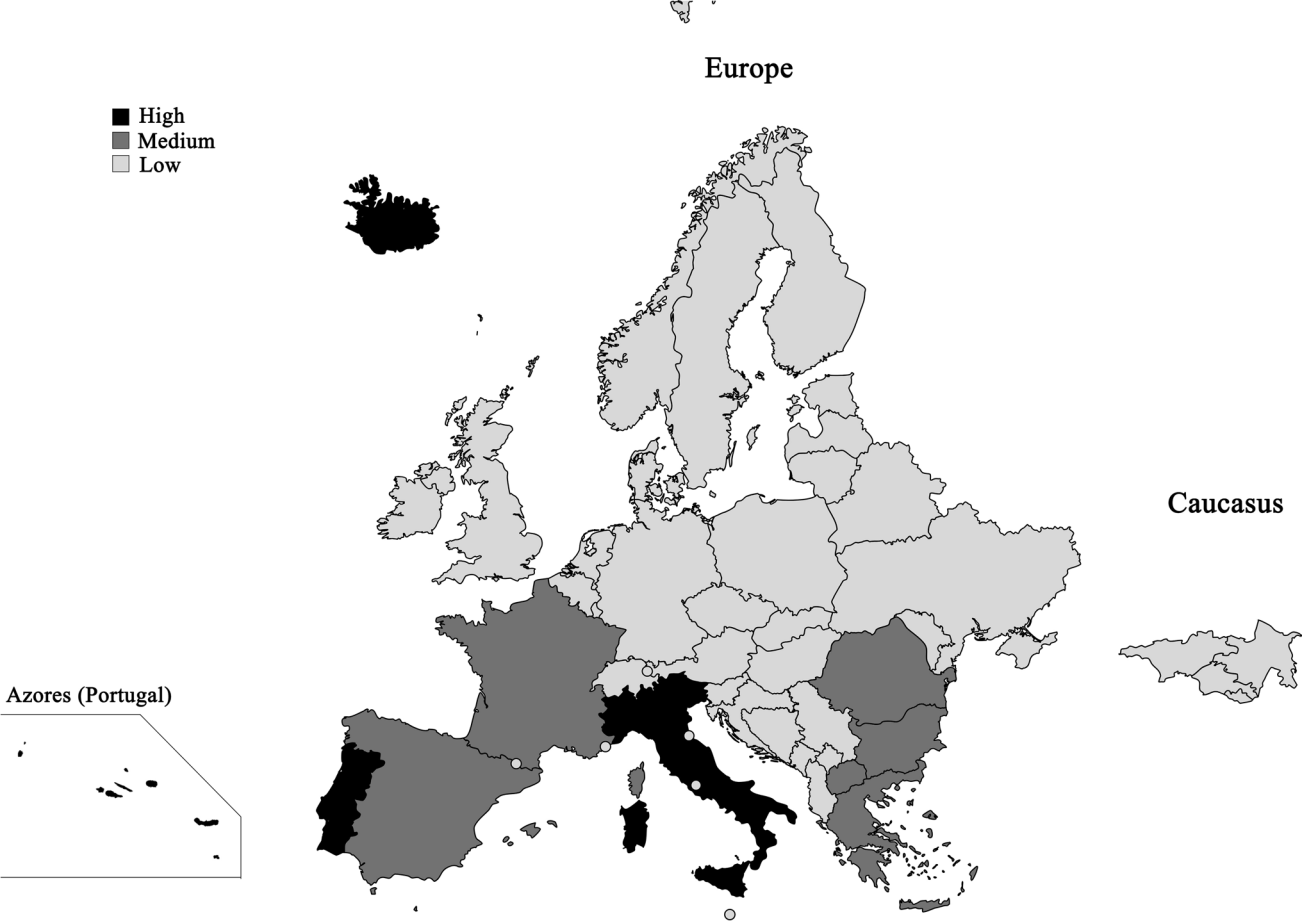
Countries in Europe classified by the number of publications about microbial and enzymatic investigations of hot springs. The classification is based on the results of literature research on sampled hot springs above 50 °C. (low: no reference to sampled hot springs was found, medium: 1–10 hot springs were found, high: > 10 hot springs were found)

Analyzed terrestrial springs with temperatures above 50 °C that harbor thermophilic microorganisms are mainly found in Southern Europe (Fig. 2). Due to the drifting of tectonic plates on the Mid-Atlantic Ridge, hotspots of terrestrial hot springs are present on Iceland and the Azores (Portugal) as well. In Eastern Europe like Romania, hot springs were mainly artificially generated by drilling holes. In other regions, geothermal springs exhibit mostly temperatures below 50 °C or no corresponding scientific publications are available so far and were, therefore, not included in this review.

Research on thermophiles in Europe was often organized in large clusters funded by the European Union. First attempts on extremophiles in general started in 1983 with the first Biotechnology program (Aguilar 1996). The base for extremophile research was created by establishing huge European network projects “Extremophiles as cell factories” and “Biotechnology of extremophiles” with more than 58 laboratories from all across the EU including isolation of novel extremophiles and their enzymes (Aguilar et al. 1998). Moreover, two large network European projects were initiated and coordinated by G. Antranikian (TUHH), which had a significant impact on the research in the field of extremophiles and their enzymes: 'Extremophiles as Cell Factories’ (58 partners including 13 industries, 1993–1996) and “Biotechnology of Extremophiles” (39 partners, 1997–1999).

The next chapters will give an overview on the research and developments of enzyme screening of Europe's hot springs as well as Caucasian hot springs sorted by regions.

## Northern Europe

Northern Europe, including Scandinavia and the British Isles, rarely harbors natural terrestrial hot springs with temperatures above 50 °C. An exception to this is Iceland, which has a wide range of hot springs owing to its ongoing emerging volcanic generation. The scientific exploration of the biodiversity of hot springs in Iceland already started in the 1970s.

## Iceland

**Fig. 3:**
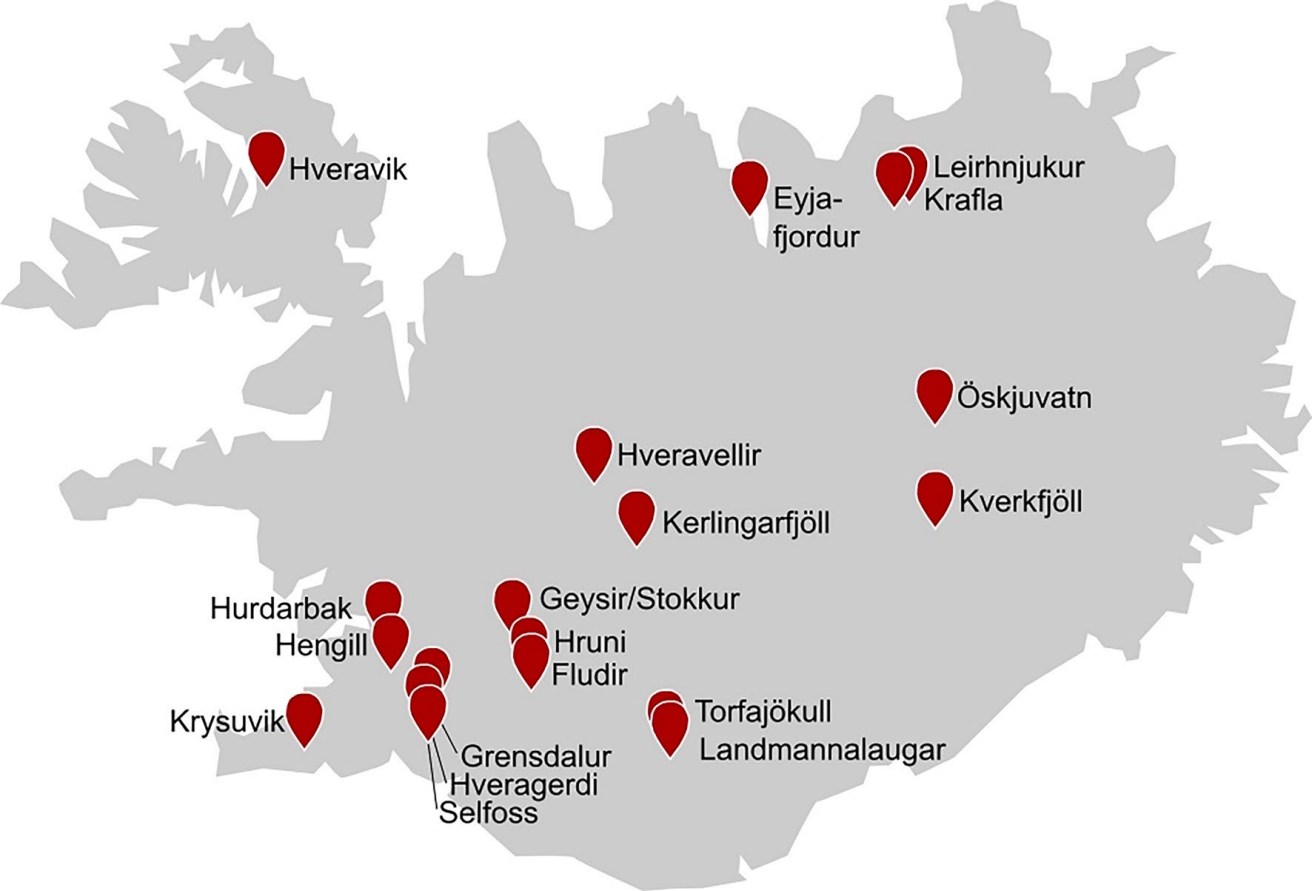
Major sampling sites of geothermal springs in Iceland

In Iceland, hot springs are spread over the whole country. They range from highly investigated terrestrial solfataric fields and alkaline springs in the vicinity of the capital Reykjavik (e.g., Hveragerði, Grensdalur, Krýsuvík) to sequestered areas like intertidal hot springs in the Isafjardardjup bay in the northwest and Kolbeinsey ridge in the north of Iceland with an island and submarine volcanoes (Fig. 3).

First microbial analysis started with the isolation and investigation of archaea from the order of Thermoproteales and Desulforococcales (Zillig et al. 1981, 1982) and bacteria from the genus of *Thermus* and *Rhodothermus* on complex medium (Pask-Hughes and Williams 1977; Kristjansson and Alfredsson 1983; Alfredsson et al. 1988). Many of the gained isolates were then further investigated for their enzyme activities. Prominent examples are isolates of *Rhodothermus marinus* derived from various sampling sites in Iceland. Examined biotechnologically relevant enzymes not only include DNA modifying enzymes but also wide-ranging polysaccharide degrading enzymes with highest activity above 85 °C (Bjornsdottir et al. 2006). To gain enzymes from *R. marinus*, different screening strategies were applied. By screening genome libraries of different *R. marinus* strains, beta-glucanase, ligase, cellulase, xylanase, and mannanase could be recovered and recombinantly produced (Spilliaert et al. 1994; Thorbjarnardóttir et al. 1995; Nordberg Karlsson et al. 1997; Halldórsdóttir et al. 1998; Politz et al. 2000). Blöndal et al. (2001) used degenerated primers for the detection of a DNA polymerase I. Based on (partial) genomic DNA sequencing and in silico identification, an enzyme with chitinase activity could be recombinantly produced (Hobel et al. 2005a). The genome of the type strain R-10^ T^ originating from Iceland was sequenced in 2009 which then gives further insights into metabolism and biotechnologically relevant enzymes (Nolan et al. 2009).

Beside *R. marinus*, many other isolated strains of Islandic hot springs were screened for enzyme activities. This includes lipases, DNA-modifying enzymes, and hydrogen production from sugars (Sigurgsladttir et al. 1993; Hjorleifsdttir et al. 1996; Hjorleifsdottir et al. 1997; Vipotnik et al. 2016). To increase the probability for identifying enzymes with desired activities, (in situ) enrichments with appropriate substrates were performed. The applied subsequent strategies to gain novel enzymes were quiet diverse. They comprise the extraction of isolates on xylan (Sonne-Hansen et al. 1993), the use of degenerated primers after enrichment with starch or chitin (Hobel et al. 2004, 2005a) or the amplification of consensus sequences and gene walking to determine neopullulanases (Labes et al. 2008; Nordberg Karlsson et al. 2008). Moreover, enrichments on glucose were carried out for the increase of high-temperature hydrogen and ethanol-producing microorganisms (Koskinen et al. 2008; Jessen and Orlygsson 2012; Jessen et al. 2015).

In parallel, sequencing technologies were developed, enabling diversity analysis of environmental samples for displaying the abundance of even unculturable species and comparison with other high-temperature sampling sites on Earth. In a high-temperature pool in Grensdalur (85–90 °C, pH 5), it was shown that a high number of sequences were annotated to uncultured species which emphasize the potential of metagenomic sequencing for the screening of novel enzymes (Menzel et al. 2015). Diverse Icelandic hot springs were explored with regard to their microbial biodiversity, including terrestrial volcanic hot springs, intertidal hot springs, and geothermal freshwater fluids on subseafloor (Marteinsson et al. 2001b, 2001a; Hobel et al. 2005b; Cousins et al. 2018). Particularly, the diversity of archaea was studied in different Icelandic hot springs investigating general archaeal diversity, nitrification or the abundance of Crenarchaeota and Korarchaeota (Kvist et al. 2007; Perevalova et al. 2008; Reigstad et al. 2008, 2010). Kvist et al. (2007) demonstrated the diversity potential in an analysis of solfataras and neutral springs in Hveragerdi. They observed variable diversity of archaea between solfatara fields but also within a single field indicates even the rich biotechnological opportunities for novel industrial enzymes. This was also confirmed by studies of Krebs et al. in 2014 and Podar et al. in 2020, which describe the influence of biodiversity by the formation of distinct niches due to temperature gradients in solfataras and alkaline hot springs of Iceland. Moreover, it was discerned that the more extreme temperature, pH, and sulfide concentration in a habitat is the lower the diversity was ascertained (Skirnisdottir et al. 2000; Menzel et al. 2015).

The sequencing technologies paved the way for a new era of sequence-based screening approaches to exploit the enzyme potential of Icelandic hot springs. Due to the high extent of these approaches, they were often organized in public funded projects running for several years. Amylomics was a EU funded project for robust enzymes applied in the carbohydrate industry (Zucko et al. 2013). Beside insights in microbial biodiversity, 4500 novel genes encoding carbohydrate active enzymes were identified by sequencing and microarray technology. High promising candidates were implemented in demonstration and marketing phase and patents. Another example is the EU FP7 Project HotZyme, a multidisciplinary approach processing culture-independent metagenomic datasets, metagenomes from enrichment cultures on polysaccharides, keratin and artificial polymers as well as isolated genomes and transcriptomes (Wohlgemuth et al. 2018; Koutsandreas et al. 2019). They developed an automated nucleotide amino acid sequences translational platform for systemic interpretation and analysis to gain novel thermostable enzymes of industrial interest, including carboxylesterases, lactonases, epoxide hydrolases, and cellulases. The huge amount of data of such projects are just at the beginning to be exploited and generate a valuable feedstock for future discovery of enzymes of particular demand.

## Central Europe

Compared to other regions in Europe, the number of thermal springs is small, often with temperatures below or slightly above 50 °C. Although they are well known and used as spas or mineral springs, nearly no scientific publications about their microbial and enzyme content are available. Hot springs with temperatures above 50 °C are, for example, located in the Rhenish Massif in Germany or in the Massif Central region in France. In the Massif Central region, a number of hot springs were already sampled to gain thermophilic isolates. From the region Chaudes-Aigues with thermal springs ranging from 40 to 82 °C, the slightly thermophilic strain *Meiothermus rufus* as well as the thermophilic bacteria *Caldinitratiruptor microaerophilus* and *Thermovenabulum gondwanense* were isolated and described (Albuquerque et al. 2009; Fardeau et al. 2010; Pradel et al. 2013). Nevertheless, there are no further investigations on the microbial community and screening of associated enzymes published so far.

## Southern Europe

In Southern Europe, many prominent hot springs are located. Geothermal areas in Portugal, with the Archipelago Azores, Italy, and Greece are particularly well investigated concerning their microbial diversity, isolates, and enzyme richness. In the Balkan region, geothermal areas are known but they are often used as spas, and little scientific research regarding thermostable enzymes or biodiversity of thermophilic microorganisms has been carried out so far. Thermostable enzymes like DNA polymerase from *Bacillus caldolyticus,* isolated from a hot spring in North Macedonia, as well as a superoxide dismutase, catalase, and oxidase from *Bacillus stearothermophilus* and *Thermotrix sp.,* isolated from a hot spring in Serbia are examples of findings from the Balkan Peninsula (Gligić et al. 2000; Popovski et al. 2017).

## Portugal and Azores

**Fig. 4:**
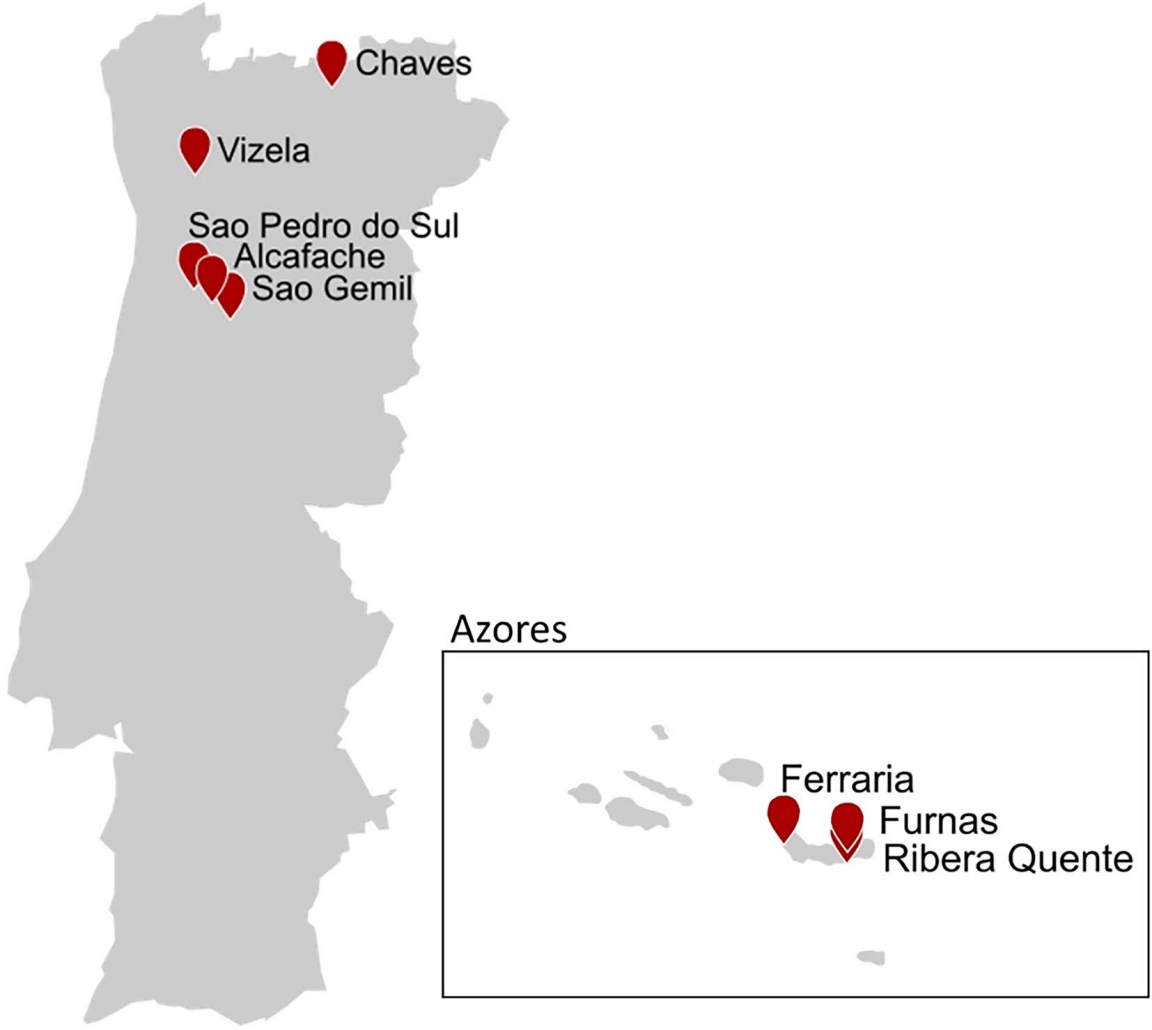
Major sampling sites of geothermal springs in Portugal and the Azores

Many hot springs in the main country of Portugal show temperatures below 50 °C used as spas. Sao Pedro do Sul (66–69 °C, pH 8–9), Alcafache (50 °C, pH 8), and Sao Gemil (48 °C, pH 8.6) are geothermal hot springs in central Portugal which have attracted the interest of microbiologists (Tenreiro et al. 1995; Ferreira Gomes et al. 2001; Alves et al. 2003) (Fig. 4). From these hot springs, several thermophilic isolates have been generated by filtering of water combined with classical plating techniques, including *Calidithermus chliarophilus, Thermus oshimai, Deinococcus murrayi, Tepidimonas* species, *Thermomonas hydrothermalis*, *Calidithermus timidus*, *Porphyrobacter cryptus* and *Raineya orbicola* (Tenreiro et al. 1995; Welch and Williams 1996; Ferreira et al. 1997; Moreira et al. 2000; Alves et al. 2003; Rainey et al. 2003; Pires et al. 2005; Albuquerque et al. 2018, 2020). *Thermus oshimai* was further analyzed for the production of a thermostable β-galactosidase (Gezgin et al. 2013). Later on, an extremely thermostable amylosucrase from *Calidithermus timidus* was identified by sequence-based screening of the genome and subsequent recombinant production and characterization (Tian et al. 2019). Nevertheless, diversity analysis or metagenomic sequencing has not been published from these habitats so far. The only approach for gaining new enzymes directly from these sampling sites was performed by Bataillon and coworkers. By plating on arabinoxylan-containing medium, a xylanolytic *Bacillus* strain could be received which produces a thermoactive xylanase with highest activity at 75 °C under the given conditions (Bataillon et al. 1998).

Another spa and hot spring region in northern Portugal is Vizela, which was less intensively sampled (vent temperature 56 °C, pH 8.9, Tenreiro et al. 1995). No further microbiological investigations have been performed so far, except for the isolation of *Meiothermus silvanus* and investigation of several *Thermus* isolates revealing a *Thermus scotoductus* strain (Santos et al. 1989; Tenreiro et al. 1995). The geothermal area of Chaves, located at the northern border of Portugal mainland is another microbial unexplored region. Although this region holds the hottest geothermal waters on the Portuguese mainland with 77 °C discharge temperature at a drilling site and furthermore a natural hot spring with 66 °C (Marques et al. 2019), no microbial investigations have been published so far.

The most prominent hot spot for geothermal springs in Portugal is the archipelago of the Azores, located on the mid-Atlantic ridge. On the main island, São Miguel, hot springs can be found at several locations including shallow marine vents at Ribera Quente and Ferraria. The most prominent terrestrial hot springs are located in the interior of the island at Furnas geothermal area (Fig. 4). In comparison to other terrestrial hot springs, metatranscriptomic analysis of filamentous mats of these hot springs revealed dominance of *Sulfurihydrogenibium azorense* which metabolizes sulfur compounds under microaerophilic conditions (Hamamura et al. 2013). In addition, analysis of thermoacidophilic Verrucomicrobia methanotrophs indicated that the isolated location of terrestrial geothermal springs like in Azores promote the allopatric speciation of microbes (Erikstad et al. 2019). Sahm et al. (2013) investigated the biodiversity of two hydrothermal springs in Furnas valley by next generation sequencing with 16S rRNA gene analysis and FISH of bacteria and archaea. Related to the results of Hamamura and coworkers, chemolithoautotrophic *Sulfurihydrogenibium* species were identified but additionally high abundance of heterotrophic prokaryotes of bacterial genera like *Caldicellulosiruptor*, *Dictyoglomus* and *Fervidobacterium* was demonstrated (Hamamura et al. 2013). A high content of organic carbon sources may have caused a natural enrichment of polymer-degrading microorganisms which are a promising source for thermostable polymer-degrading enzymes (Sahm et al. 2013).

Further investigations underlined the capacity for obtaining novel biotechnologically relevant enzymes. Enrichment cultures with spent coffee ground and its different polymeric components revealed remarkable shifts in the community composition depending on the substrate indicating substrate-specialized genera (Suleiman et al. 2019). Earlier enrichments on feathers have already revealed keratinase-producing strains (Friedrich and Antranikian 1996; Riessen and Antranikian 2001). Many isolates from the Azores have been substrate-independently gained mainly by the group of Da Costa, including slightly thermophilic strains (Albuquerque et al. 2012) and *Thermus* isolates (Manaia and da Costa 1991). Based on isolated strains of the Azores, restriction enzymes and a xylanase were identified (Marques et al. 1998; Welch et al. 1998). In addition, genome analysis of *Truepera radiovictrix* and *Sulfurihydrogenibium azorense* resulted in the discovery of an amylosucrase (Zhu et al. 2018) and the most catalytically effective alpha-carbonic anhydrase investigated so far (De Luca et al. 2013).

Other approaches for identification of novel biotechnologically relevant enzymes were based on the activity-based screening of metagenomic libraries. Schröder and coworkers identified an archaeal beta-glucosidase in an *E. coli* hosted library (Schröder et al. 2014). Remarkably, the thermophilic screening host *Thermus thermophilus* BL03 provides an effective alternative for successful screening for esterases from hot springs compared to conventional *E. coli* screening host (Leis et al. 2015).

Moreover, sequence-based screening of metagenomes opens new possibilities for host-independent screening. By this, thermostable enzymes like an alpha-galactosidase and a laminarinase were obtained from metagenomic datasets of Azorean hot springs (Schröder et al. 2017; Burkhardt et al. 2019). The probability to find high-active substrate-specific enzymes was further increased by a multi-omic screening approach. Using anaerobic enrichments in the presence of green coffee beans, metagenomic and metatranscriptomic screening approaches identified 14 promising hydrolase-coding genes. Four of them were recombinantly expressed and the enzymes were characterized as active thermostable endoglucanase, endomannanase or beta-glucosidase (Busch et al. 2021). This demonstrates that multi-omic approaches are efficient tools for the identification of novel active enzymes from hot spring environments.

## Spain

Spain has a series of hot springs with temperatures below 50 °C distributed throughout the country. These are mainly used as spas.

**Fig. 5:**
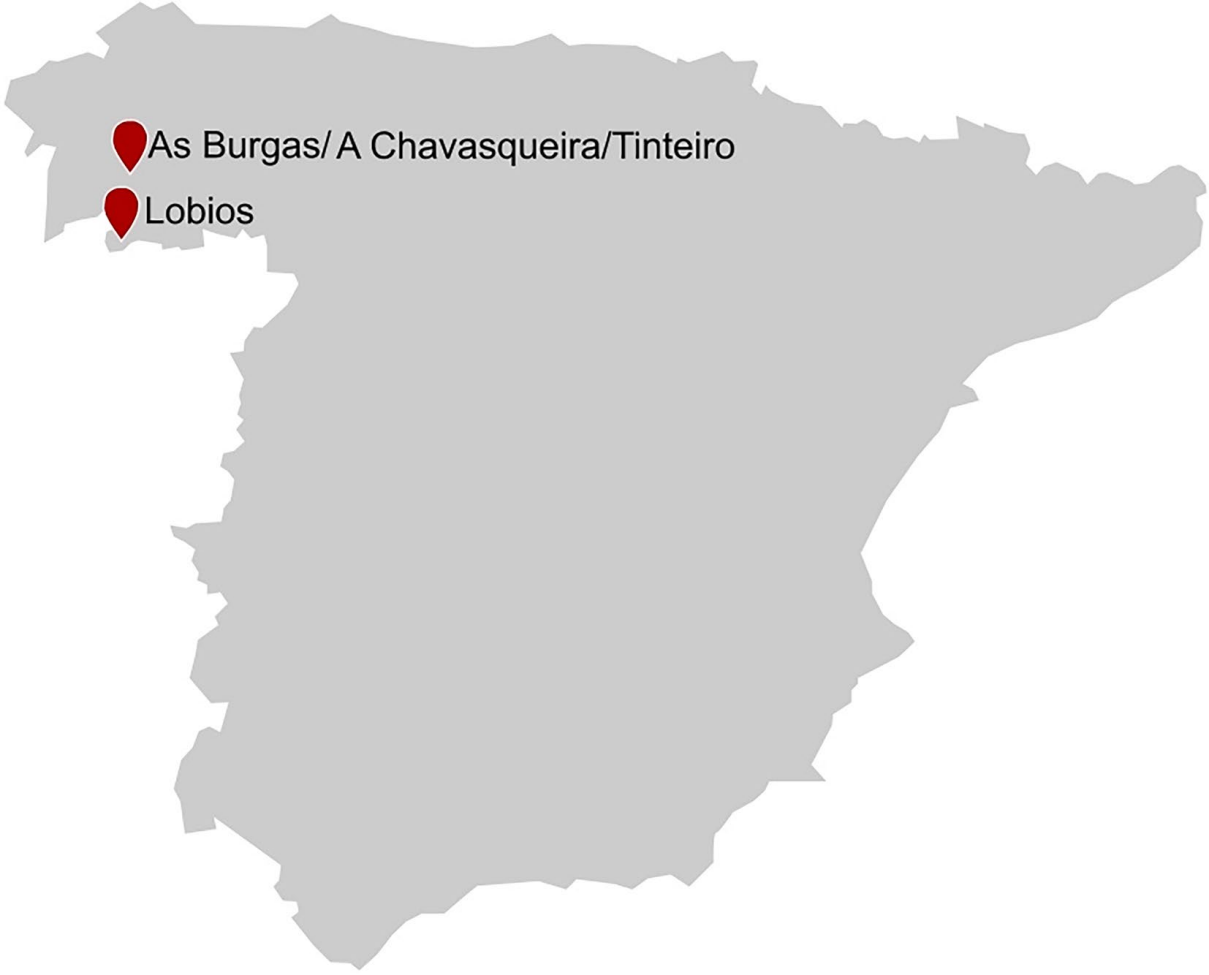
Location of the hot springs As Burgas, A Chavasqueira, Tinteiro and Lobios in Spain

The region of Galicia in the North-West of Spain is rich in geothermal springs. Besides a large number of thermal springs with temperature below 50 °C, hot springs like As Burgas, Lobios, A Chavasqueira, and Tinteiro in the province of Ourense show temperatures above 50 °C (Fig. 5). Several of these hot springs have been investigated as potential sources for enzymes from thermophilic microorganisms.

In 2013, Deive and colleagues analyzed the abovementioned hot springs to isolate lipolytic enzyme-producing thermophilic microorganisms from thermal spots, where biodiversity has never been investigated so far (Deive et al. 2013). With agar plate cultures, they identified 12 strains with potential lipolytic activity and, moreover, nine of them were described as true lipase producers. In addition, using submerged culture techniques, two strains from A Chavasqueira hot spring, CH6A and CH6B, showed remarkable extracellular enzyme activity. 16S rRNA gene sequencing revealed highest homology with *Bacillus* sp. and *Anoxybacillus gonensis*.

In 2015 and 2019, the working group EXPRELA from the university of A Coruña analyzed Lobios hot spring toward microbial diversity and screened for novel hydrolytic enzymes (López-López et al. 2015; Knapik et al. 2019). Lobios hot spring is characterized by water temperatures up to 76 °C and slightly alkaline pH 8.2. Both biodiversity studies revealed that bacteria were predominant domains with 61% and 93%, respectively. López-López et al. (2015) used sequence-based screening and functional screening of a generated fosmid library in parallel to identify six genes encoding for lipolytic activity in total. The most interesting candidate LOB4Est was heterologously expressed in a mesophilic yeast host and biochemically characterized.

For the identification of xylan-degrading enzymes, Knapik and coworkers compared open reading frames (ORFs) with BLAST and the CAZy database to retrieve potential xylanase-encoding ORFs (Lombard et al. 2014). By functional screening with an established metagenomic fosmid library comprising approximately 150.000 clones, a xylanase-encoding gene *XynA3* was identified and later expressed in *E. coli* BL21 (Knapik et al. 2019).

DeCastro and coworkers utilized a similar approach for the identification of thermostable beta-galactosidases from the microbial community inhabiting As Burgas geothermal spring. By Illumina sequencing, it was shown that bacteria dominated the microbial community (93%) with Proteobacteria and Aquificae being the most abundant phyla, while archaea (6%) and eukaryotes (0.67%) were in the minority. Two ORFs which were similar to GH2 beta-galactosidase genes were found in the assembled reads of which one showed activity after recombinant expression. The enzyme exhibited β-galactosidase activity and high thermal stability (DeCastro et al. 2021).

## Italy

**Fig. 6:**
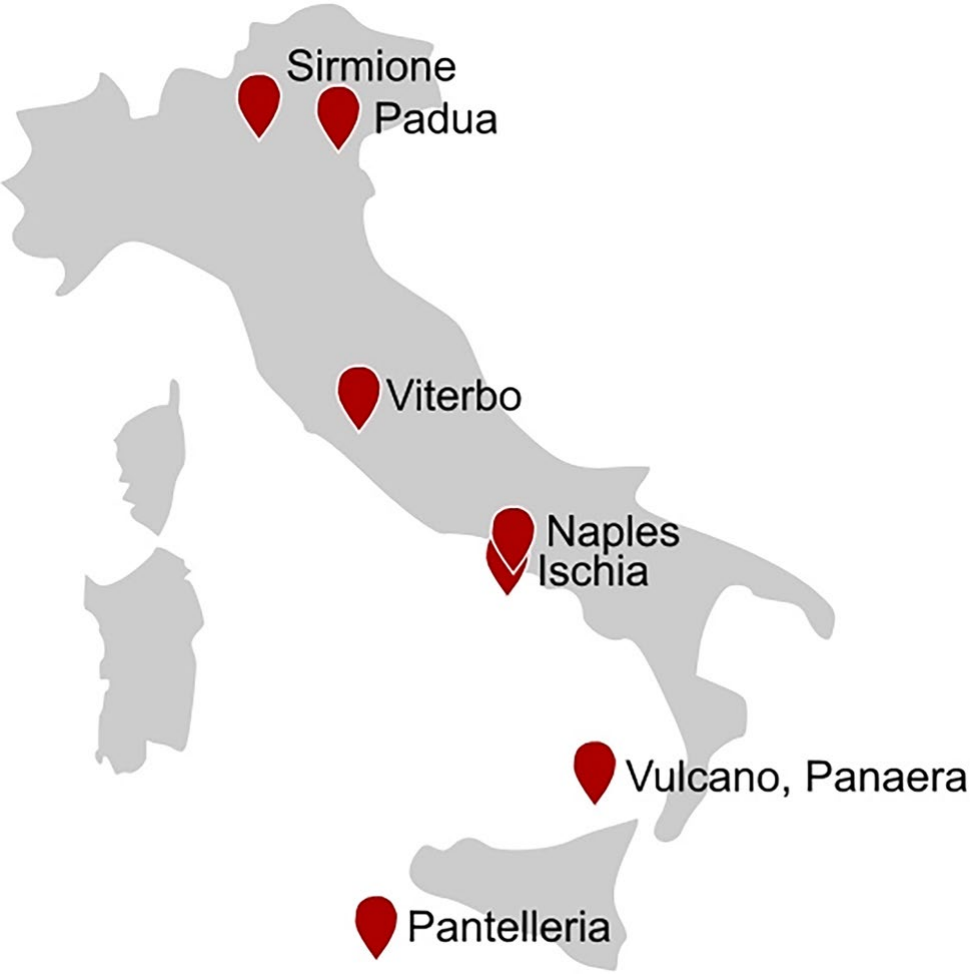
Major sampling sites of geothermal springs in Italy

Geothermal hot springs with thermophilic microorganisms and temperatures above 50 °C are spread over the whole country (Minissale 1991) (Fig. 6). Many of them are used as natural spas. The best microbially examined spring in northern Italy is an Euganean hot spring in the area of Padua, called Abano. Although it reaches temperatures of up to 75 °C, so far microbial diversity was only analyzed from thermal muds of around 50 °C with special focus on cyanobacteria (Gris et al. 2020). However, from earlier samples, several type strains have been isolated indicating a rich thermophilic microbial community with potential biotechnological relevant enzymes. This includes *Fervidobacterium pennivorans* with pullulanase I activity (Koch et al. 1997). The pullulanase-encoding gene was subsequently obtained by screening of a genomic plasmid library from *F. pennivorans* (Bertoldo et al. 1999). Moreover, *Thermoanaerobacter italicus* which produces thermostable pectate lyases and *Anoxybacillus thermarum* with potential glycoside hydrolases and glycoside transferases were obtained from Abano (Kozianowski et al. 1997; Poli et al. 2015). Another hot spring in northern Italy which attracted the interest of microbiologists is a spa in Sirmione on a peninsula of lake Garda. With thermal waters of meteoric origin of up to 69 °C, the microbial community is mainly composed of sulphur-cycling bacteria and has not been used for screening of thermoactive enzymes so far (Paduano et al. 2018).

On the western coast of the central part, the majority of Italy’s hot springs are concentrated. Since Etruscian and Roman civilization, Viterbo hydrothermal area in the surrounding of Rome has been known and used as spas. The different alkaline springs have a large chemical and temperature range between 30 and 60 °C (Piscopo et al. 2006). In 1995, the microbial investigation of six hydrothermal sites started by the isolation of thermophilic amylolytic and pullulolytic *Bacillus* strains and anaerobic bacteria known later as *Thermoanaerobacter thermohydrosulfuricus* (Canganella and Trovatelli 1995). Later on, the biodiversity of Bullicame hot spring was investigated culture dependent (plate-based isolation) and culture independent (16S rRNA gene sequencing) and showed a higher biodiversity in the microbial mats compared to the water samples which was composed of aerobic and anaerobic heterotrophs as well as phototrophs (Valeriani et al; 2018). Moreover, from Bullicame hot spring, the thermophilic *Caloramator viterbensis* was isolated and described (Seyfried et al. 2002).

One hot spot for hot springs in Italy is the area of Naples harboring multiple terrestrial geothermal hot springs, solfatara, and shallow marine vents around the city as well as on Ischia Island (Fig. 6). Menzel et al. analyzed the microbial diversity of two terrestrial hot springs in the area of Pisciarelli (86 °C, pH 5.5) and Pozzuoli (76 °C, pH 3.0). The microbial composition of both samples differs as Pisciarelli was dominated by archaea from *Acidianus* and *Pyrobaculum* species whereas Pozzuoli was mainly dominated by bacteria of the genus *Acidithiobacillus*. (Menzel et al. 2015). Metagenomic sequences of Pozzuoli samples were also used for in silico screening of thermostable amine transferases (Ferrandi et al. 2017). Additionally, two hydrothermal mud/water pools of the solfatara Pisciarelli with temperatures of 85 and 92 °C and acidic conditions of pH 5.5 and 1.5 were later on investigated by Strazzulli and coworkers (Strazzulli et al. 2020). The datasets of the metagenomic analysis could be nearly exclusively assigned to sequences of archaeal origin which was also observed by Menzel et al. (2015). However, the pools in this study were dominated by species of the *Sulfolobales* order whereby interestingly up to two-thirds of the reads had no match to known sequences in the NCBI database (Strazzulli et al. 2020). Functional classification of the ORFs revealed the highest amount for carbohydrate metabolism. The remarkably high number of carbohydrate active enzymes including glycoside hydrolases, polysaccharide lyases, and carbohydrate esterases may be caused by rich vegetation around the hot springs (Strazzulli et al. 2017). Altogether, the findings exhibit these hot springs as valuable resources for novel thermostable enzymes of biotechnological interest. Moreover, many microbial isolates were gained from the Pisciarelli area which are interesting candidates for biotechnological applications. One famous example is *Saccharolobus solfataricus* also known as *Sulfolobus solfataricus* from which many different biotechnologically relevant enzymes were screened by cultivation experiments or in silico genome analysis including thermostable polymerase, proteases, esterase/lipases, aldolase, lactonase, and various glycoside hydrolases (McDonald et al. 2006; Rémy et al. 2016; Quehenberger et al. 2017; Curci et al. 2021). Another interesting example is the discovery of a new α-glucosidase from an unknown hyperthermophilic archaeon characterized by a temperature optimum of 95 °C by a combination of a culture-dependent approach and metagenomics (Iacono et al. 2022).

However, due to geothermal activity, the Pisciarelli region is subject to ongoing changes influencing the microbial community composition by temperature, pH, and in situ chemistry. Iacono and coworkers analyzed three newly formed sampling sites and demonstrated their differences in biodiversity and composition of carbohydrate active enzymes indicating the unexplored potential of this area (Iacono et al. 2020). The influence of earthquakes on the microbial community was also the subject of a study on Ischia Island with its manyfold hot springs next to Naples. Although the autochthone thermophilic community of the hot spring was augmented by bacteria from soil, sediments, sea, freshwater, and wastewaters, the conditions have not changed and a natural restoration process to the original community was observed after a few months (Valeriani et al. 2020).

One of the best examined microbial hot spots in whole Europe is Vulcano Island as part of the Aeolian archipelago in the southern part of Italy. For decades, microbiologists have analyzed the thermophilic microorganisms of the hydrothermal system and gained many thermophilic marine isolates. This resulted in an impressive number of isolates. One of the best studied hyperthermophilic species is *Pyrococcus furiosus* which type strain Vc 1 was isolated already in the 1980s from a submarine solfataric field in a depth between 2 and 10 m (Fiala and Stetter 1986). Many other isolates of the order Thermococcales were gained in the following years from the shallow marine vents of Vulcano Island. The enzyme potential of Thermococcales is huge and many thermostable enzymes have been characterized for biotechnological application fields exhausting a large portion of it. An extensive overview about these DNA polymerases, carbohydrate and protein hydrolases and many other enzymes relevant for biocatalysis is given by Schut et al. (2014). Methods for enzyme identification from Thermococcales isolates range from classical activity-based screening of genomic libraries in *E. coli* to restriction-independent cloning of a gene expression library based on the genome sequence of *P. furiosus* (Yuan et al. 2015).

Another industrial relevant isolate gained from geothermally heated marine sediments from Vulcano Island is the type strain *Thermotoga maritima* MSB8, which produces enzymes for the degradation of diverse polysaccharides (Conners et al. 2006). Among all thermophilic genomes sequenced so far, the genome of *T.* *maritima* possesses with 7% the highest proportion of glycoside hydrolases (Chhabra et al. 2002; Sadaqat et al. 2021). As for many other isolates, genome sequencing of *T. maritima* has opened a new era for sequence-based enzyme discovery, for example coming up with thermostable esterase, fructose-1,6-bisphosphatase, and cellulase/mannanase (Levisson et al. 2007; Myung et al. 2010; Sadaqat et al. 2021).

After the great era of gaining microbial isolates from Vulcano Island, the focus switched to the exploration of the microbial consortia to investigate even unculturable microorganisms. As already expected from the huge amounts of isolated strains, metagenomic analyses confirmed the rich biodiversity of thermophilic microorganisms in the area of Vulcano Island. The microbial analysis of La Fozza crater with fumaroles and comparison with other sampling sites at Vulcano Island indicated a potential correlation between the microbiome and the emitted gas composition (Fagorzi et al. 2019). However, the best studied site of Vulcano Island is the hydrothermal area in Levante Bay which was investigated at multiple sites by next generation sequencing (Antranikian et al. 2017; Fagorzi et al. 2019). Moreover, enrichment cultures on cellulose were performed and revealed a shifted community with high abundance of archaea and the presence of *Paleococcus* and *Thermococcus* species compared to the original samples (Antranikian et al. 2017). Surprisingly, the abundance of *Thermotoga* species which could not be detected in the original sample underlines the relevance of enrichment cultures for gaining substrate-specific enzymes from environmental samples. A culture-independent screening approach for novel enzymes was performed by Placido and coworkers. From metagenomic DNA of a shallow marine vent in Levante Bay, a fosmid library was constructed followed by an activity-based screening for lipases/esterases/lactamases, haloalkane and haloacid dehalogenases and glycoside hydrolases. The evaluation of positive clones revealed 200 enzyme-encoding genes with high biotechnological potential and elucidates the enzymatic diversity which is far from being fully explored (Placido et al. 2015).

Even on the neighboring island of the Aeolian archipelago, hydrothermal vents are present. Maugeri and coworkers analyzed two vents on Panarea Island and came upon a complex microbial community composed of phototrophs and chemolithotrophs. Attempts to investigate the enzyme potential have not been done yet (Maugeri et al. 2009). Furthermore, Pantelleria Island in the very south of Italy is harboring the fumarolic field of Favara Grande with temperatures of up to 111.6 °C. Two sampling sites were compared by next generation sequencing. Although the two sites were closely neighbored, their microbial communities differed significantly (Gagliano et al. 2016). These results demonstrate the microbial variety and enzyme potential for biotechnological applications of even closely neighbored sampling sites.

## Greece

Due to the subduction of the African plate under the Eurasian plate, Greece harbors a variety of geothermal areas, most of them located along the coastal areas or in the form of shallow marine vents, and on islands in the Aegean Sea (Duriez et al. 2008).

**Fig. 7:**
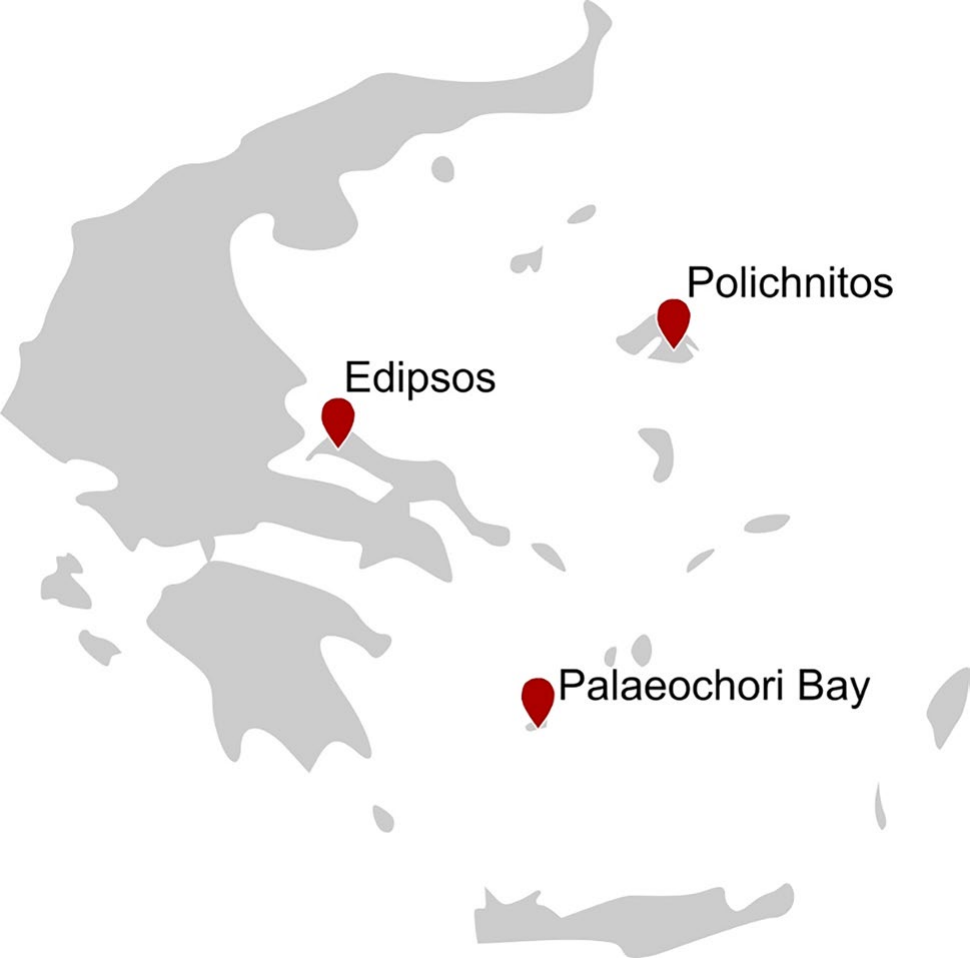
Location of geothermal areas and hot springs in Greece

Shallow marine vents provide a variety of thermal gradients and are rich in nutrients and minerals, located, among other sites, in the Palaeochori Bay in the south-east of the island Milos in the Aegean Sea (Fig. 7). First analyses of thermophilic bacterial consortia around shallow marine vents in the Palaeochori Bay started in 1999 with a cultivation-based approach and molecular methods such as DGGE analysis of amplified 16S rRNA gene fragments (Sievert et al. 1999). The first isolated microorganisms of said shallow marine vent system were the hyperthermophilic archaea *Thermococcus aegaeicu*s and *Staphylothermus hellenicus* growing optimally at 90 °C and 85 °C, respectively (Arab et al. 2000). Furthermore, Sievert and Kuever isolated the thermophilic sulfate-reducing bacterium *Desulfacium hydrothermale* from heated sediment of the same shallow marine vent system (Sievert and Kuever 2000).

Greece is a country rich in terrestrial hot springs, although most exhibit temperatures below 50 °C. The Polichnitos hot spring on the island of Lesvos has been investigated for its microbial diversity at temperatures of up to 89 °C (Kormas et al. 2009; Mizerakis et al. 2017). The archaeal community consisted of only two phylotypes, Crenarchaeota (93.9% dominance) and *Archaeoglobus fulgidus*, while the most abundant bacterial phylotype was identified as *Hydrogenophilus thermoluteolus* (Kormas et al. 2009). At the outflow of Polichnitos hot spring, 16S rRNA gene analysis revealed bacterial species commonly detected in hot springs (Mizerakis et al. 2017). However, strains related to species never observed in hot environments but in marine habitats were identified as well, indicating a unique bacterial community. Bacterial and archaeal diversity have also been investigated at Edipsos hot spring by sequencing the 16S rRNA encoding genes, resulting in the dominant archaeal phylotype Crenarchaeota (43.2%) and dominant bacterial phylotype *Persephonella hydrogeniphila* (61.9%) (Kormas et al. 2009).

Besides microbial diversity analyses of shallow marine vents and a few hot springs, research targeted more toward novel biocatalysts from these communities has not been performed until now. With its richness of microbial diversity, Greece harbors great potential for further investigation when it comes to extremozymes.

## Eastern Europe

Eastern Europe harbors many geothermal areas, often used as spas or energy sources. Except for Bulgaria and Romania, for the majority of these hot springs to our knowledge, no publication about the microbial diversity or potential biotechnologically relevant strains and enzymes is available. Hence, Eastern Europe still harbors a great potential for the discovery of new industrially relevant biocatalysts.

## Bulgaria

Over 700 springs from about 140 natural deposits can be found in Bulgaria, with cold water springs in the North and warm or hot water springs located more toward the South of the country**.** The hyperthermal mineral waters in the Rhodopes mountains and in the valley of the rivers Struma and Mesta, the big Rupi basin (94.2 ha) and 56 hot springs around Velingrad are among the most well-known terrestrial hot water sources in Bulgaria (Kambourova 2018). With more than 80 registered thermal springs of different geotectonic origin and physico-chemical properties, Bulgaria shows great potential for the discovery of novel thermophilic microorganisms and enzymes for industrial application (Ivanova et al. 2011).

**Fig. 8:**
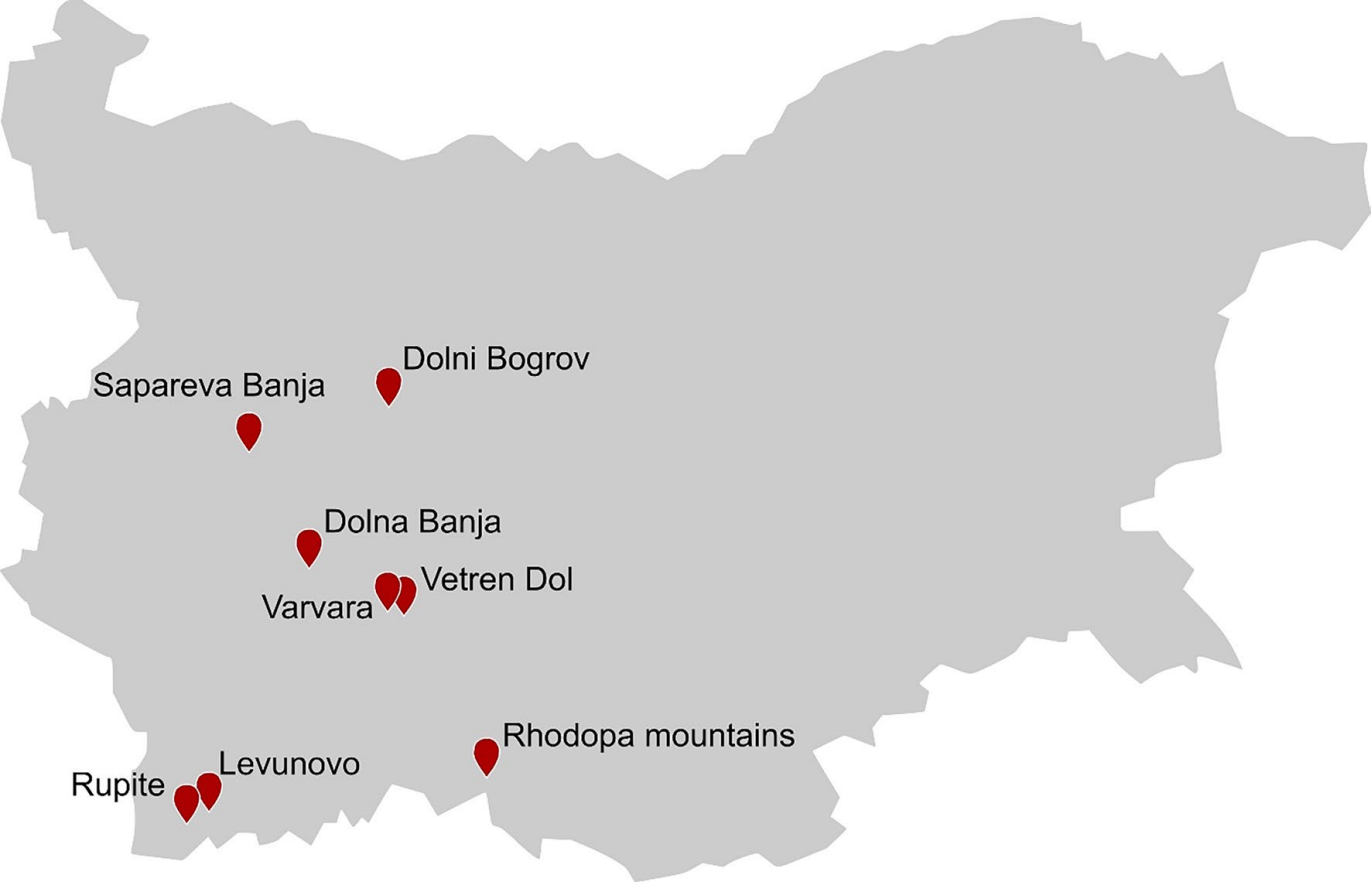
Major sampling sites of geothermal areas and hot springs in Bulgaria

The starting point for research on thermophiles from Bulgarian hot springs was set in 1993 by the isolation of several thermophilic *Bacillus* strains with lipase or xylanase activity from samples of a hot spring in South-West Bulgaria Fig. 8. With *Anoxybacillus rupiensis* and *Anoxybacillus bogrovensis* isolated from the Rupi basin and Dolni Bogrov, further novel thermophilic bacteria were characterized in the early 2000s (Derekova et al. 2007; Atanassova et al. 2008).

In 2011, attention shifted more toward microbial diversity, and hence the unseen potential of hot springs in Bulgaria, since only a small fraction of microorganisms can be cultivated in the laboratory. While some studies focused primarily on archaeal diversity, others were studying the diversity of bacterial and archaeal consortia altogether (Ivanova et al. 2011; Stefanova et al. 2015; Strunecký et al. 2019). With a culture-independent approach, archaeal diversity of a hot spring near the city Valvara in South-East Bulgaria was investigated by 16S rRNA gene analysis. 35 archaeal operational taxonomic units (OTUs) were detected, belonging to three archaeal phyla with the majority (23) affiliating to Crenarchaeota. The bacterial and archaeal diversity in two geographically separated hot springs Levunovo and Vetren Dol, South Bulgaria, was investigated by Stefanova and coworkers by sequencing the 16S rRNA and GH-57 genes of the samples. The archaeal diversity was shown to be significantly higher in the hotter spring Levunovo (82 °C) with 28 sequence types belonging to five archaeal groups from Crenarchaeota and Euryarchaeota, while bacterial diversity was higher in the hot spring Vetren Dol (68 °C) (Stefanova et al. 2015).

Thermophilic isolates were specifically chosen for their ability to metabolize specific carbon sources such as xylan, gellan, and other carbohydrates, since at temperatures above 60 °C carbohydrate solubility is high and viscosity of the reaction mixture is reduced (Emanuilova et al. 2000; Derekova et al. 2006, 2008). The first approach was to screen samples from hot springs for microorganisms with a specific enzymatic activity through cultivation-based methods. Emanuilova and coworkers found lipase, as well as xylanase producing *Bacillus* sp. using this approach (Emanuilova et al. 1993, 2000). Derekova and coworkers were using samples from various hot springs in Bulgaria for diversity analyses and screening for carbohydrases simultaneously (Derekova et al. 2008). A gellan lyase and a superoxide dismutase were produced by *Geobacillus stearothermophilus* from Sapareva banja hot spring and *Bacillus licheniformis* from Dolna banja hot spring, respectively (Derekova et al. 2006; Boyadzhieva et al. 2010).

Bulgaria is a country rich in geothermal areas that have been investigated for diversity and valuable enzyme producing isolates as well. Nevertheless, further investigation utilizing sequence-based approaches in combination with the already used culture-based approaches can bring out the full potential of these microbial communities and their enzymes.

## Romania

In the Western Plain of Romania, over 500 drillings were made between the 1960–1980s in the search of thermo-mineral springs that could be further used as an energy source (Coman et al. 2011). The resulting drilling sites harbor a great abundance of biomats, and thus microbial diversity.

In 2011 and 2013, Coman and coworkers investigated the microbial diversity of different biomats from drilling sites in Romania. With a cultivation-based approach, the cyanobacterial diversity from a drilling site in Marghita with a temperature of 67 °C was determined (Coman et al. 2011) Fig. 9. This resulted in eight cyanobacterial taxa with Oscillatoriales, Phormidium, and Leptolyngbya being the most abundant. Another drilling site with a temperature of 55 °C was studied using a similar approach. 16S rDNA gene libraries were created and sequenced. Only a low diversity of archaea was observed with three represented taxa *Methanomethylovorans thermophila*, *Methanomassiliicoccus luminyensis*, and *Methanococcus aeolicus*, while bacterial diversity was higher, with Cyanobacteria, Chloroflexi, and Proteobacteria being dominant (Coman et al. 2013). From the Romania hot spring Tășnad, the thermophile *Anoxybacillus flavithermus* was isolated and its esterase/lipase was biochemically characterized (Chiş et al. 2013).

**Fig. 9:**
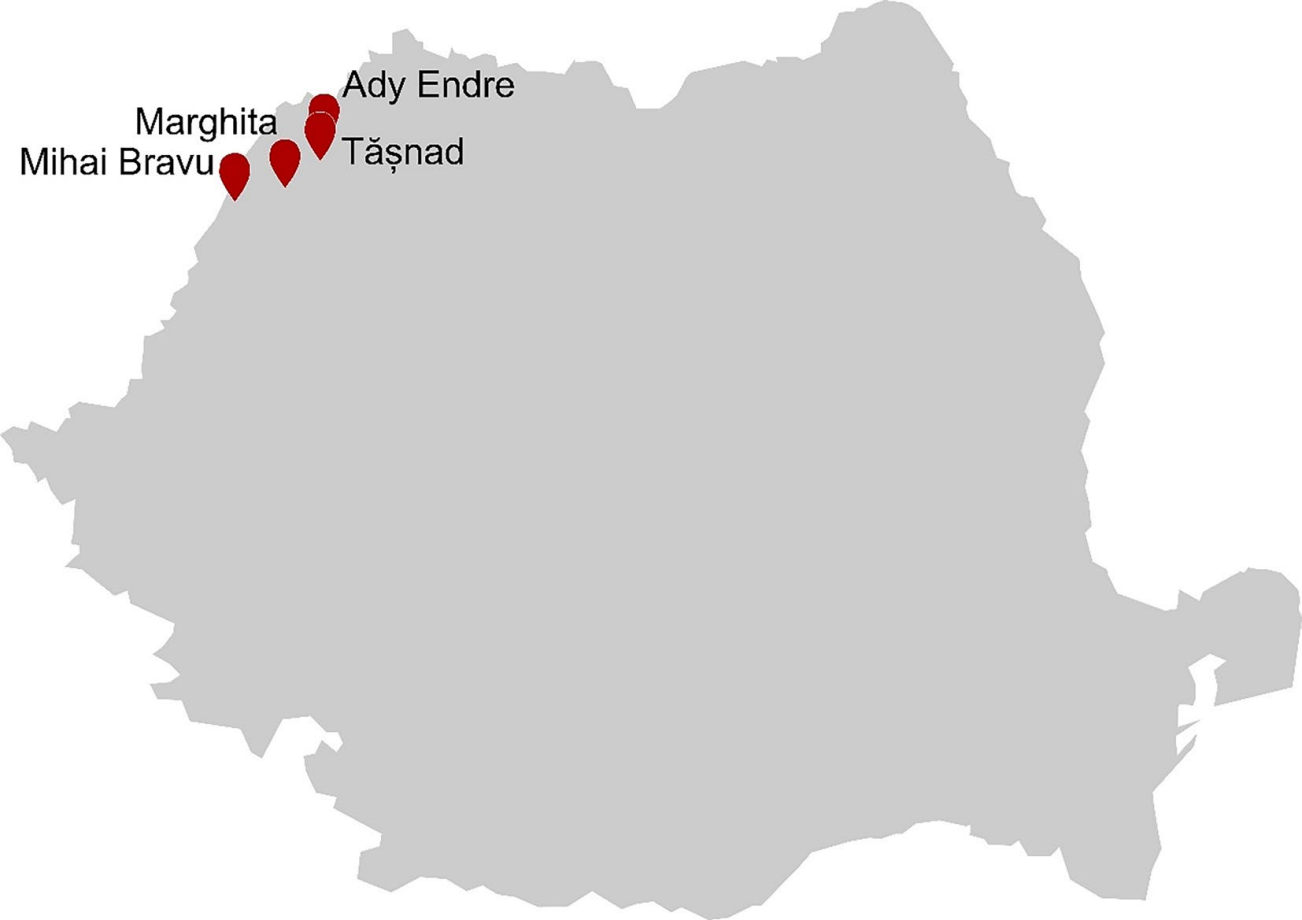
Major sampling sites of geothermal areas in Romania

Romania is not as rich in natural terrestrial hot springs as other European countries, but harbors geothermal areas that were used for energy production. These sites can be further investigated to deepen the understanding of its microbial diversity and use of biocatalysts for industrial applications.

## Caucasus region

The Caucasus region is located between Europe and Asia, and it is known for its rich culture, diverse landscapes, and natural hot springs. There are many hot springs located throughout the region, each with its unique features and benefits. The Caucasus Mountains include the Greater Caucasus in the north and Lesser Caucasus in the south. The Caucasus Mountains formed largely as the result of a tectonic plate collision between the Arabian plate moving northwards with respect to the Eurasian plate (Reilinger et al. 1997). The Caucasus Mountains are part of seismically active Alpine-Himalayan orogenic belt and has been a center of significant volcanic activity during the Quaternary period, which led to the formation of the number of high altitude (> 600 m above sea level) and mineralized geothermal springs with different geochemical properties (Karakhanian et al. 1997; Henneberger et al. 2000). Some of the studied springs have been identified as prospective sources of geothermal energy and are commercially usable for agriculture, space heating, hot water supply, and tourism (often used as spas) (Henneberger et al. 2000). The most famous hot springs of the Caucasus region are Tbilisi Sulphur Baths in Georgia, Jermuk hot springs in Armenia, Karvachar hot springs in Nagorno-Karabakh, Ganja hot springs in Azerbaijan, and Karmadon hot springs in North Ossetia-Alania, Russia.

The hot springs in the Caucasus region are known for their unique microbial diversity. The first investigation on thermophiles from Caucasian hot springs was set in 1990s by the isolation of several thermophilic *Bacillus* and *Thermus* strains from samples of hot springs located in Karabakh Plateau (Akhmedova; 1991). Recently the microbiome of several Caucasian geothermal hot springs were published (Guliyeva et al. 2015; Panosyan et al.; 2018a; Saghatelyan et al. 2021a; Geliashvili et al. 2021; Toshchakov et al. 2021). Despite these previous efforts, a comprehensive census of the microbial communities in whole Caucasus region is still lacking.

## Armenia and Nagorno-Karabakh

**Fig. 10:**
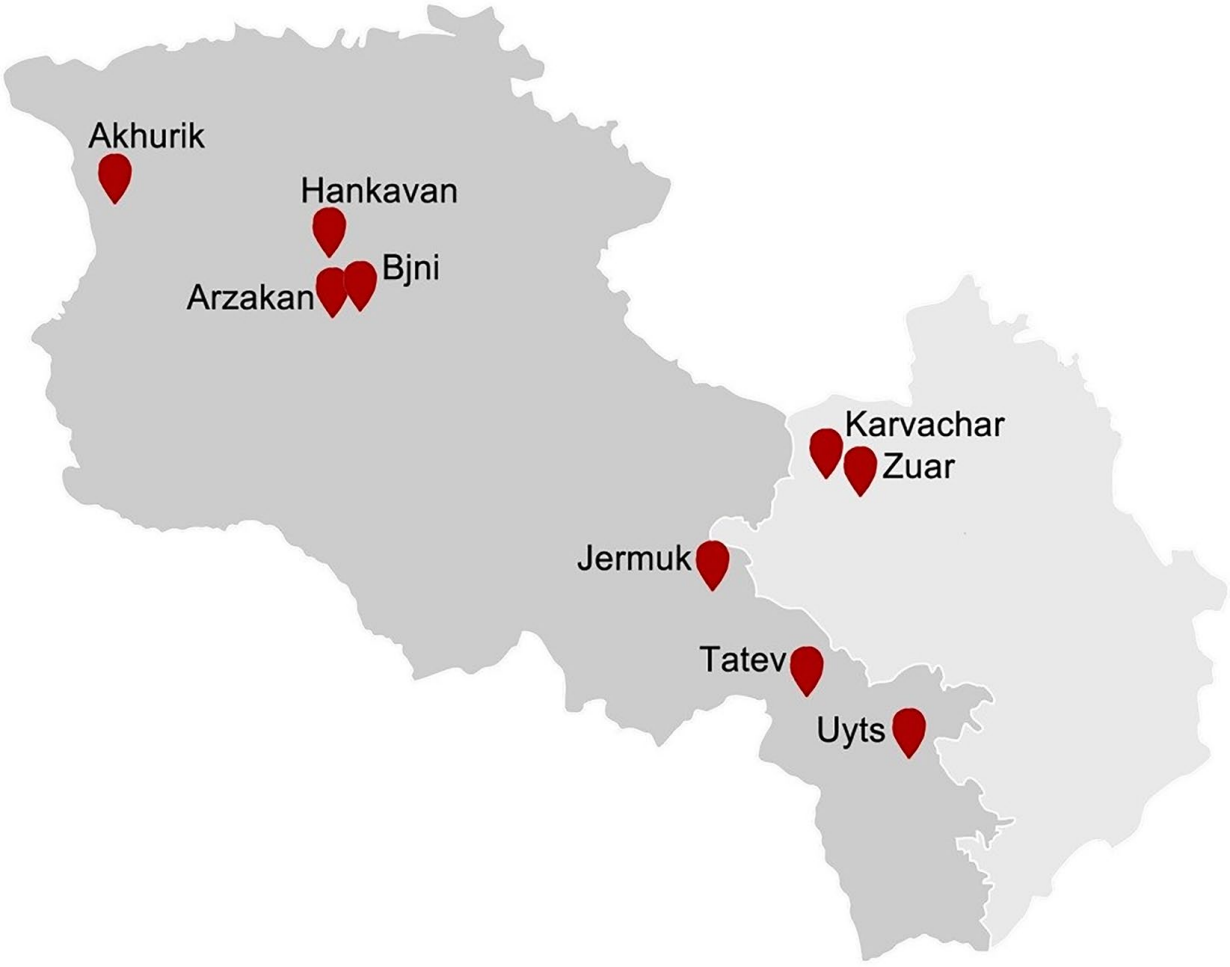
Major sampling sites of geothermal springs in Armenia (grey) and Nagorno Karabakh (light grey)

On the territory of Armenia, numerous geothermal springs with different geotectonic origins are found (Henneberger et al. 2000). In Armenian hot springs, the temperature varies from 25.8 to ≥ 64 °C, pH value is neutral, moderately alkaline, or alkaline in nature and have mainly mixed cation–mixed anion compositions (Panosyan et al. 2018a, b). The highest temperatures are measured in Jermuk (64 °C), Arzakan (44 °C), Hankavan (42 °C), Bjni (37 °C), and Sayat-Nova (36 °C) hydrothermal systems (Fig. 10). One of the most famous hot springs known for its natural beauty and clean air is the Jermuk hot spring located in the town of Jermuk.

Nagorno-Karabakh is located in the southeastern part of the Lesser Caucasus. It is typically mountainous, embracing the eastern part of the Karabakh Plateau with the Artsakh valley, forming the great part of the Kura-Araks lowland. Numerous geothermal springs at high elevations with different physicochemical properties are found also on the territory of Nagorno-Karabakh (Panosyan et al. 2018a). Geothermal springs found on the territory of Nagorno-Karabakh are also mainly classified as springs with moderate temperature. Two of Nagorno-Karabakh geothermal springs located in Karvachar (≥ 70 °C) and Zuar (42 °C) are characterized with higher water temperature.

A majority of the hot springs found in Armenia and Nagorno-Karabakh are anthropogenically influenced and often used by tourists and local people for bath. Some of the geothermal springs like Jermuk and Karvachar hot springs are used for balneology for curing rheumatismal and dermatological as well as digestive disorders and physical exhaustion (Panosyan et al. 2018a).

Overall, the hot springs in the Caucasus region are a unique and fascinating natural resource.

During the past decades, the phylogenetic diversity of microbial community thriving in Armenian and Nagorno Karabakhian geothermal springs has been explored following both cultivation-based and culture-independent approaches. The studies of taxonomic diversity of hot spring microbiomes examined using DGGE, 16S rRNA gene library construction, 454 pyrosequencing, and Illumina HiSeq have revealed abundance of bacterial phyla Proteobacteria, Bacteroidetes, Cyanobacteria, and Firmicutes. Archaea mainly include the phyla Euryarchaeota, Crenarchaeota, and Thaumarchaeota, and comprise less than 1% of the prokaryotic community (Panosyan and Birkeland 2014; Panosyan et al. 2017, 2018a; Hedlund et al. 2013). The microbial diversity of Karvachar hot spring was represented by phyla Proteobacteria, Bacteroidetes, Actinobacteria, Deinococcus-Thermus (Saghatelyan et al. 2021a).

Several cultivation-based studies have been performed to describe novel strains/species derived in Armenian geothermal springs. Many thermophilic microbes as producers of exopolysaccharide (EPS) and thermozymes like lipase, protease, amylase, and DNA polymerase have been isolated from targeted geothermal springs. More than 22 distinct species belonging to the eight genera *Aeribacillus, Anoxybacillus, Bacillus, Brevibacillus, Geobacillus, Parageobacillus, Paenibacillus*, and *Ureibacillus* were identified (Panosyan et al. 2020). Many isolated bacilli shared 91–97% sequence identity with their closest matches in GenBank, indicating that they belonged to novel taxa, at least at the species level. 71% of the isolates were found to actively produce at least one or more extracellular protease, amylase, or lipase (Shahinyan et al. 2017; Panosyan et al. 2020; Tadevosyan et al. 2022). Two active producers of EPSs, *Geobacillus thermodenitrificans* ArzA-6 and *Geobacillus* (*Parageobacillus*) *toebii* ArzA-8 have been isolated from Arzakan hot springs (Panosyan et al. 2018b).

A thermophilic spirochete *Treponema* sp. J25 was isolated from Jermuk geothermal spring. It showed only 95.1% 16S rRNA sequence similarity with *Treponema caldarium* H1^T^, indicating that strain J25 potentially represents a novel species in the genus *Treponema*. Genomic analyses further confirmed that *Treponema* sp. J25 is the only known thermophilic free-living treponeme with metabolic potential for nitrogen fixation (Poghosyan 2015).

Two aerobic thermoactinomycetes with growth T_opt_ 50–55 °C and pH_opt_ 7.0–7.4 able to produce extracellular hydrolases were isolated from Akhurik and Tatev geothermal springs (Panosyan 2019).

A gammaproteobacterial methanotroph, *Methylocaldum* sp. AK-K6 strain was isolated from Akhurik geothermal spring and identified based on both phenotypic and phylogenetic characteristics (Islam et al. 2015).

Sediment samples from Arzakan and Jermuk geothermal springs were used to enrich nitrite-oxidizing bacteria (NOB) at 45–50 °C. It was shown that *Nitrospira calida* and *Nitrospira moscoviensis* were the dominant species in examined samples (Edwards et al. 2013). Acetoclastic and hydrogenotrophic methanogenic enrichment at 45 °C and 55 °C using sediments from Jermuk and Arzakan geothermal springs also were obtained (Hedlund et al. 2013; Panosyan and Birkeland 2014).

Thermophilic keratinolitic bacteria were isolated from water and sediments samples of Arzakan, Karvachar, and Zuar geothermal springs by accumulation cultures using a chicken feather as a source of carbon and nitrogen. The strains were identified as *B. licheniformis, B. borbori* and *Thermobacillus* sp., and able to hydrolyze feather after 96 h of incubation, at 55 °C (Tadevosyan et al. 2022).

A new species of thermophilic bacterium belonging to the genus *Anoxybacillus* named *Anoxybacillus karvacharensis* sp. nov. (= DSM 106524 T = KCTC 15807 T) has been discovered from Karvachar hot spring (Panosyan et al. 2021).

Recently a novel strain of *Thermus scotoductus* (designated K1) was isolated from sludge samples of Karvachar hot spring. The DNA polymerase (TsK1) obtained from the thermophile *T. scotoductus* K1 has the potential to be commercialized. The better base insertion fidelity of TsK1 DNA polymerase is a feature that demonstrates an advantage over *Taq* DNA polymerase and can be used in various high-temperature polymerization reactions (Saghatelyan et al. 2021b). Recently, metagenomic library was obtained from Jermuk hot spring. Sequence-based screening of metagenomic datasets let to two characterized carbohydrate active enzymes. A Jermuk-lamM gene (accesion number OK490392) encodes a putative GH family 16 laminarinase and a Jermuk-celP encodes a putative GH family 5 cellulase. Jermuk-LamM is a novel 1,3-β-glucanase as it shares only 69% amino acid sequence similarity to the endo-1,3-beta-glucosidase available in the NCBI database. For today, this is the only characterized endo-1,3-β-D-glucanase in *Marinmicrobia* genus. The product of the gene Jermuk-CelP is a novel 1,4-β-glucanase showing the highest 49.1% identity with the biochemically characterized endo-β-1,4-endoglucanase of *Thermotoga maritima*. Both enzymes have similar temperature and pH optima, being active at temperature range of 45–60 °C and pH range of 5–7. Both enzymes showed resistances to various additives and metal ions, thus are suitable candidates for a wide range of industrial applications including the production of biofuels and pharmaceuticals (Paloyan et al. 2022).

Overall, the microbial diversity of the geothermal springs in Armenian and Nagorno-Karabakh is incredibly rich and diverse, and there are many different species and strains that have yet to be fully characterized. The study of these microorganisms is an important area of research that has the potential to yield insights into the evolution of life on Earth, as well as to develop new biotechnologies and medical treatments.

## Georgia

**Fig. 11:**
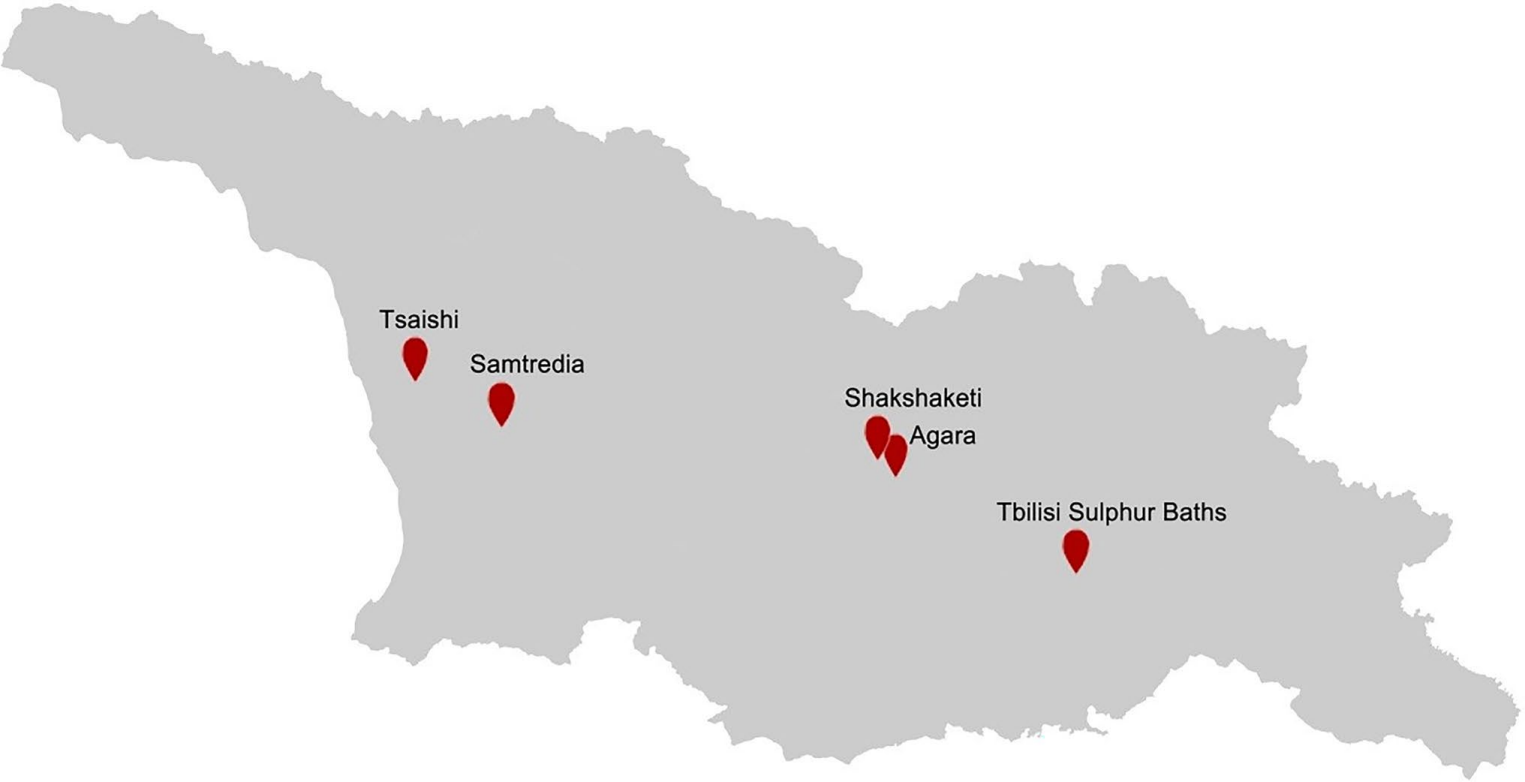
Major sampling sites of geothermal hot springs in Georgia

Georgia is located between the Greater Caucasus in the north and the Lesser Caucasus range in the South, and has considerable resources of natural thermal waters. Over 250 natural thermal springs and artificial wells as well as spring clusters are known with water temperatures ranging from 30 to 108 °C. Mesothermal springs (with outlet temperature 30–35 °C) are mainly found in Borjomi, Tsikhisjvari, Tskaltubo, and Saberio areas, while hot springs (with outlet temperature > 78 °C) are found in Zugdidi-Tsaishi, Kvaloni, and Kindgi regions (Fig. 11). The drilled wells are mainly used in balneology and as a thermal source for greenhouses (Melikadze et al. 2010). The Georgian geothermal springs are characterized by diverse chemical composition, with mineralization ranging from 0.2 mg/L (Borjomi region) to 11.3 mg/L (Aspindza region). The Georgian thermal waters have mixed cation and mixed anion ratios mainly composed of hydrocarbonate, chloride, sulfate, sodium, potassium, magnesium, and calcium ions. All studied springs are rich in heavy metals and majority of them contain gasses such as hydrogen sulfide, methane, nitrogen, and carbon dioxide (Melikadze et al. 2010).

The investigation of microbial community structure in Georgian geothermal springs has been explored recently following culture-independent approaches. The sequence reads from the Samtredia geothermal spring (Georgia) water sample with an outlet temperature of 58 °C and pH 7.15, obtained from the whole-genome shotgun sequencing on Illumina HiSeq 2500 platform, showed that majority of bacterial sequence reads were affiliated with the Firmicutes and Gammaproteobacteria, followed by Actinobacteria, Betaproteobacteria, Alphaproteobacteria, Chlamydia, and Bacteroidetes. Archaeal sequence reads were affiliated with Crenarchaeota and Euryarchaeota (Panosyan et al. 2018a).

Microbial assemblages of the second studied spring, Tsaishi sediment, were dominated by Deinococcus-Thermus, Actinobacteria, and Aquificae. Zugdidi-Tsaishi hot spring is characterized by an outlet temperature of 82–90 °C and pH 6.0 (Geliashvili et al. 2021). One of the most famous hot springs in the Caucasus region is the Tbilisi Sulphur Baths in Georgia. These baths have been a popular destination for centuries, and they are renowned for their healing properties. The water is naturally heated and infused with minerals like sulfur, which is believed to have therapeutic effects on the body.

The microbial diversity of the Tbilisi sulfur spring (Georgia) was analyzed using whole-genome shotgun sequencing using Illumina MiSeq platform. The sequences obtained from metagenomic DNA showed exclusive prevalence of Proteobacteria, followed by minor phyla such as, Nitrospirae, Firmicutes, Bacteroidetes, and Chloroflexi archaeal sequence reads were also in the minority, belonging to the Euryarchaeota and comprising < 1% of total reads (Panosyan et al. 2018a).

At the genus level, sulfur-oxidizing bacteria of genus *Sulfurihydrogenibium* and anaerobic thermophilic bacteria of genus *Ammonifex*, capable to reduce nitrate to ammonium prevailed in the Samtredia, whereas the Tbilisi Sulfur spring was highly represented by chemoorganotrophic thermophilic community, belonging to *Acidovorax* and *Polaromonas* (Panosyan et al. 2018b; Geliashvili et al. 2021).

Three novel thermophile bacteria were isolated from water and mud samples of hot spring in the central Georgia villages Shakshaketi and Agara where temperatures around 82 °C are measurable (Vardigoreli et al. 2005). According to biochemical and 16S rRNA gene sequence analysis, isolated strains were identified as *Fervidobacterium* sp., *Caloramator australicus* and *Pseudothermotoga lettingae*. An anaerobic strain *Fervidobacterium* sp. GSH with optimal growth at 75 °C showed high ability to degrade keratin (native chicken feathers), which is a challenge for the current field of biotechnology. By following a multi-omics approach, its proteolytic system was explored, and new keratinase encoding genes were cloned (Geliashvili et al. 2021). Subsystem-based functional annotation approach suggested high prevalence of genes associated with clustering-based subsystems, protein metabolism, amino acids and its derivatives, and carbohydrates metabolism in both springs. The genes for secondary metabolism and metabolism of aromatic compounds have also been detected, but with higher representation in the Tbilisi Sulfur Spring, indicating possible presence of genes of biotechnological importance in the studied thermal springs.

## North Ossetia-Alania, Russia

North Ossetia-Alania is located in the southern part of the North Caucasus and separated from Georgia and South Ossetia by Greater Caucasus Range. On the territory of North Ossetia-Alania, a number of subterranean thermal reservoirs and terrestrial geothermal springs were found (Zaalishvili et al. 2015). These habitats are characterized mainly by moderate temperature (55 °C) and mineralization ranging from 1.1 to 2.1 g/L. Thermal water of the sources is used by local people as a thermal spa.

Recently microbial diversity of those springs has been investigated. In total, representatives of 50 phyla were detected with Bacteria having total dominance over Archaea. The most abundant phyla were Proteobacteria, Firmicutes, and Bacteroidota which represented ~ 70 to 80% of the communities. The microbial community of the hot springs is significantly influenced by anthropogenic factors but is dominated by moderate thermophiles (Toshchakov et al. 2021).

## Conclusion

The potential for the discovery of unique microorganisms and novel enzymes of thermophilic origin is still a long way from being exhausted. The majority of thermostable enzymes that have been isolated and characterized from European terrestrial hot springs and geothermal areas have been derived from Bacteria or Archaea. Culture-independent approaches like metagenomics and other “-omics” show great potential to close the gap between the unknown and unculturable microorganisms to unveil the immense magnitude of potential enzyme variants.

Even though the major hot spots in Europe such as Iceland, the Azores, and Italy have been well investigated, utilizing metagenomic analyses methods and microbial isolates, their manifold sequence data still comprise an unused treasure of enzymes. Furthermore, there is still a high number of lesser known and unexplored hot springs above 50 °C, harboring a great potential for undiscovered thermostable enzymes and microbial diversity to confront the ever-growing demand for the development of future sustainable bio-based industry.
